# CNTNAP4 deficiency in dopaminergic neurons initiates parkinsonian phenotypes

**DOI:** 10.7150/thno.40798

**Published:** 2020-02-10

**Authors:** Wenlong Zhang, Miaomiao Zhou, Weiye Lu, Junwei Gong, Feng Gao, Yuanquan Li, Xuandong Xu, Yuwan Lin, Xiaokang Zhang, Liuyan Ding, Zhiling Zhang, Guihua Li, Xiang Chen, Xiangdong Sun, Xiaoqin Zhu, Pingyi Xu, Yunlong Zhang

**Affiliations:** 1 Department of Neurology, The First Affiliated Hospital of Guangzhou Medical University, Guangzhou 510120, China.; 2 Key Laboratory of Neurological Function and Health, School of Basic Medical Sciences, Guangzhou Medical University, Guangzhou 511436, China.; 3 Information center of Guangzhou Power Supply Bureau Co., Ltd., Guangzhou 510620, China.; 4 The First Affiliated Hospital of Gannan Medical University, Ganzhou, Jiangxi, China.; 5 School of Basic Medical Sciences, Second Affiliated Hospital of Guangzhou Medical University, Guangzhou 510260, China.; 6 School of Basic Medical Sciences, Guangzhou Medical University, Guangzhou 511436, China.; 7 Shenzhen Research Institute of Xiamen University, Shenzhen 518000, China.

**Keywords:** Parkinson's disease, CNTNAP4, dopaminergic neurons, α-synuclein, mitophagy

## Abstract

**Rationale**: Contactin-associated protein-like 4 (CNTNAP4) belongs to the neurexin superfamily and has critical functions in neurological development and synaptic function. Loss of CNTNAP4 in interneurons has been linked to autism, schizophrenia, and epilepsy. CNTNAP4 is also highly enriched in dopaminergic (DA) neurons in the substantia nigra (SN), however, few studies have investigated the role of CNTNAP4 in DA neurons, and whether CNTNAP4 deficiency in DA neurons contributes to Parkinson's disease (PD) remains unclear.

**Methods**: Effects of CNTNAP4 knockdown or overexpression on the DA MN9D cell line were assessed via Western blotting, immunocytochemistry, and RNA sequencing. An *in vivo* animal model, including CNTNAP4 knockout mice and stereotaxic injections of adeno-associated viral short-hairpin RNA with the tyrosine-hydroxylase promotor to silence CNTNAP4 in the SN, as well as the resulting physiological/behavioral effects, were evaluated via behavioral tests, Western blotting, immunohistochemistry, and transmission electron microscopy. Enzyme-linked immunosorbent assays (ELISAs) were performed to examine the cerebrospinal fluid (CSF) and plasma CNTNAP4 concentrations in PD patients.

**Results**: We demonstrated that CNTNAP4 knockdown induced mitophagy and increased α-synuclein expression in MN9D cells. CNTNAP4 knockdown in the SN induced PD-like increases in SN-specific α-synuclein expression, DA neuronal degeneration, and motor dysfunction in mice. In addition, CNTNAP4 knockdown in SN-DA neurons increased autophagosomes and reduced synaptic vesicles in the SN. Furthermore, CNTNAP4 knockout mice showed movement deficits, nigral DA degeneration, and increased autophagy, which were consistent with the SN-specific knockdown model. We also found that CSF and plasma CNTNAP4 expression was increased in PD patients; in particular, plasma CNTNAP4 was increased in male PD patients compared with controls or female PD patients.

**Conclusion**: Our findings suggest that CNTNAP4 deficiency may initiate phenotypes relevant to PD, of which we elucidated some of the underlying mechanisms.

## Introduction

Parkinson's disease (PD) is a common age-related neurodegenerative disease. Clinically, PD is mainly characterized by motor dysfunctions that include bradykinesia, resting tremors, gait disturbances, and rigidity 1. PD pathologically manifests as abnormal deposits of α-synuclein aggregates and chronic death of dopaminergic (DA) neurons in the substantia nigra (SN) 2, 3. The etiology and pathogenesis of PD are considered to comprise a complex interplay of aging, genetic susceptibility, and environmental factors 4. Genetic susceptibility plays an important role in PD pathogenesis, and highly relevant genes include those encoding α-synuclein, leucine-rich repeat kinase 2, glucocerebrosidase, parkin, PTEN-induced kinase 1, and DJ-1^5^-^7^. However, despite this knowledge about PD susceptibility, the pathogenesis of PD is still not completely clear.

It is well known that dysfunctional synaptic transmission (e.g., DA, GABAergic, and glutamatergic) mediated by aberrant synaptic cell-adhesion molecules, such as those from the neurexin family, plays an important role in the pathogenesis of neurodegenerative disease, including PD and Alzheimer's disease (AD)^8^-^10^. Contactin-associated protein-like 4 (CNTNAP4) (encoded by *Cntnap4*/*Caspr4*) belongs to the neurexin superfamily and has critical functions in neuronal cell-cell interactions, which are important for neural development and synaptic function. CNTNAP4 is uniquely expressed in the central nervous system, and like other neurexins, has a short cytoplasmic domain that contains a carboxy-terminal binding site for PDZ domains 11. In addition, CNTNAP4 is highly enriched in developing murine interneurons and interacts with presynaptic proteins such as Mint1 and calcium/calmodulin-dependent serine protein kinase, which are important for synaptic function and GABAergic synaptic transmission 12, 13. Increasing evidence supports the fact that CNTNAP4 may be related to autism, childhood-onset schizophrenia, and epilepsy, highlighting the possible important role that CNTNAP4 may have in neurological development 14-17. Recently, Karayannis *et al.* 18 reported that CNTNAP4 contributes to GABAergic synaptic transmission and CNTNAP4 knockout mice display autistic-like behavior. Moreover, CNTNAP4 affects neuron excitability and inhibits synaptic transmission through postsynaptic GABA type-A receptors (GABA_A_Rs), and CNTNAP4 influences GABA_A_R transport and membrane expression through regulating GABA_A_R-associated protein (GABARAP) 17. CNTNAP4 has also been reported to be highly enriched in the SN and ventral tegmental area (VTA) of the midbrain, which—aside from containing GABAergic neurons—also contain DA projection neurons 11, 18. In addition, the vast majority of tyrosine hydroxylase (TH)-positive DA neurons also express CNTNAP4, and the loss of CNTNAP4 results in failures in neurotransmission at DA synapses 18. Hence, the localization of CNTNAP4 and DA synaptic phenotypes in the absence of CNTNAP4 suggest that CNTNAP4 may play specific roles in DA synaptic function. Although it has been suggested that the CNTNAP4 gene and its intronic copy number variation (CNV) polymorphism are associated with aging and aging-related disease, such as PD and AD in females 19, the role of CNTNAP4 in neurodegenerative disease remains unclear.

In this study, for the first time, we demonstrated a novel role of CNTNAP4 in DA neurons *in vitro* and *in vivo*, and we suggest that CNTNAP4 deficiency may initiate parkinsonian phenotypes relevant to PD. Importantly, loss of CNTNAP4 specifically in SN-DA neurons resulted in pathophysiological changes consistent with those in PD, such as increased α-synuclein expression and DA neuronal degeneration in the SN. Moreover, we elucidated that the underlying mechanisms of PD-like phenotypes following loss of CNTNAP4 involve induction of mitophagy and disruption of synaptic function. Intriguingly, we found that CNTNAP4 expression in cerebrospinal fluid (CSF) and plasma was increased in PD patients compared with that of controls. Hence, our findings suggest that CNTNAP4 may represent a promising therapeutic target in PD patients.

## Materials and Methods

### Reagents

MPTP (1-methyl-4-phenyl-1,2,3,6-tetrahydro pyridine) and MPP^+^ (1-methyl-4-phenylpyridinium-iodide) were purchased from Sigma-Aldrich (St. Louis, MO, USA). Anti-TH (F-11, sc-25269), dopamine transporter (DAT, sc-32258), α-synuclein (sc-69977), GABA_A_Rβ3 (sc-376252), microtubule-associated 2 (MAP-2, sc-74422), and pyruvate dehydrogenase E1α (PDH-E1α) (sc-377092) antibodies were purchased from Santa Cruz Biotechnology (Dallas, TX, USA). Anti-synapsin І (#5297), α-synuclein (#4179), synaptotagmin (#14558), syntaxin (#18572), GABARAP (#13733), postsynaptic density protein 95 (PSD-95, #3450) and synaptosomal nerve-associated protein 25 (SNAP-25, #5308) antibodies were purchased from Cell Signaling Technology (Danvers, MA, USA). Anti-microtubule-associated protein-1 light chain 3 (LC3) II/І (ab48394), p62 (ab5416), GABA_A_Rα2 (ab193311), and ubiquitin (ab7780) antibodies were purchased from Abcam (Cambridge, MA, USA). Anti-CNTNAP4 (bs-11076R-2) and CNTNAP4 (orb544737) antibodies were purchased from Bioss (Beijing, China) and Biorbyt LLC (San Francisco, CA, USA), respectively. Anti-GABA_A_Rα1 (3108661) was purchased from Millipore (Billerica, MA, USA). Anti-NDP52 (12229-1-AP) and GAPDH (60004-1) antibodies were purchased from Proteintech Group (Rosemont, IL, USA). DyLight 488 goat anti-mouse IgG (H+L) (70-GAM4882) and DyLight 594 goat anti-rabbit IgG (H+L) (70-GAR5942) were purchased from Multi Sciences (Hangzhou, China). Horseradish peroxidase (HRP)-labeled goat anti-rabbit IgG and HRP-labeled goat anti-mouse IgG were purchased from Beyotime Biotechnology (Shanghai, China). EGFP-vector and EGFP-CNTNAP4 plasmids were constructed by Dongze Biotechnology Co., Ltd. (Guangzhou, China). The Mitochondrial Membrane Potential Assay Kit with JC-1 was purchased from Beyotime Biotechnology (Shanghai, China).

### Animals

The 8-week-old C57BL/6 male mice (25 ± 2 g) used for CNTNAP4 adeno-associated virus (AAV)-short hairpin RNA (shRNA) virus injection were purchased from SPF Biotechnology Co., Ltd. (Beijing, China). *Cntnap4* knockout mouse model used in this study was designed and developed by Shanghai Model Organisms Center, Inc (Shanghai, China). Briefly, Cas9 mRNA was *in vitro* transcribed with mMESSAGE mMACHINE T7 Ultra Kit (Ambion, TX, USA) according to the manufacturer's instructions. Four single guide RNAs (sgRNAs) targeted to delete exon 3 of *Cntnap4 gene* (sgRNA1: TGCCACTTGTGTTCATTTA GAGG; sgRNA2: TGCCTCTAAAT GAA CACAA GTGG; sgRNA3: ATGGTTTAGT GGACTCGTGTGGG; sgRNA4: CATGGTTTAGTGGACTCGTGTGG) were *in vitro* transcribed using the MEGAshortscript Kit (ThermoFisher, USA).* In vitro*-transcribed Cas9 mRNA and sgRNAs were then injected into zygotes of C57BL/6J mouse, and transferred to pseudopregnant recipients. Obtained F0 mice were validated by PCR and sequencing using primer pairs: F-5'-CCAAACCCAATTCATTCCTT-3'; R-5'-GCAACACTGTAAATCACGCATA-3'. The positive F0 mice were chosen and crossed with C57BL/6J mice to obtain F1 heterozygous* Cntnap4* knockout mice. The genotype of F1 mice was identified by PCR and confirmed by sequencing. Male and female F1 heterozygous mice were intercrossed to produce the homozygous *Cntnap4* knockout mice. Male *Cntnap4* knockout mice (nearly 12 weeks) were used in this study, and wild-type (WT) littermates were set as the control. Three to four mice were kept in each cage under a controlled 12/12-h light/dark cycle, temperature (22 ± 1°C), relative humidity (60 ± 5%), and food and water were provided *ad libitum*. The experiment was started one week after the mice were acclimated. All the experiments were conducted according to the National Institute of Health guidelines on the care and use of animals (NIH Publications No. 8023, revised 1978) and were approved by the Institutional Animal Care and Use Committee of Guangzhou Medical University.

### Dopaminergic MN9D cell cultures

MN9D cells were purchased from American Type Culture Collection (Manassas, VA, ATCC) and were cultured with Roswell Park Memorial Institute (RPMI) 1640 Medium containing 8% fetal bovine serum (Invitrogen, Carlsbad, CA, USA), 1% 200 U/mL penicillin (Beyotime Biotechnology), and 1% 200 mg/mL streptomycin (Beyotime Biotechnology) at 37°C under 5% CO_2_ air in an incubator.

### Primary neuronal cultures

As described previously 20, pregnant C57BL/6 mice were anaesthetized at 16-18 days of gestation, after which embryonic mice were obtained. Briefly, the midbrain of each brain was collected, cut into sections, 2 mL of 0.25% trypsin was added, and sections were digested in an incubator at 37°C for 30 min. Single cells were collected and plated at 2.5 × 10^6^ cells/well in six-well plates that were coated with poly-l-lysine (0.33 mg/mL). After the cells adhered to the plate, DMEM/F12+2% B27 culture medium was added for three days. Additionally, 2.5 µg/mL cytosine culture medium was added to inhibit the growth of glial cells and other hybrid cells in order to obtain primary neurons. MAP-2 labeling was used for immunofluorescence detection of primary neurons.

### Drug treatments

A stock solution of MPP^+^ was prepared in 0.01 M of phosphate-buffered saline (PBS) and the stock concentration of MPP^+^ was 1 mM. Then, MN9D cells were treated with 50, 100, 200, or 500 µM MPP^+^ for 24 h, whereas controls were treated with PBS. To examine CNTNAP4 expression in different PD animal models, subacute and chronic MPTP models were constructed according to the study of Jackson-Lewis and Przedborski^21^. Mice in the subacute group were intraperitoneally injected with MPTP at 30 mg/kg once a day for five days, while the control group was injected with saline. Midbrain and striatal samples in each group were collected on day 3 after the first MPTP injection (named 3 d), day 3 after the last MPTP injection (named 5+3 d), and day 8 after the last MPTP injection (named 5+8 d). Mice in the chronic group were intraperitoneally injected with MPTP at 25 mg/kg twice a week for a total of five weeks, while the control group was injected with saline. Midbrain and striatal samples in each group were collected after five weeks of MPTP administration.

### Cellular viability assay

A cell counting kit-8 (CCK-8) was used to measure MN9D cellular viability according to our previous study 22. After treating with 50, 100, 200 or 500 µM of MPP^+^ for 24 h, MN9D cells were subjected to the CCK-8 assay according to the manufacturer's instructions. Briefly, culture medium was removed, and plates were washed twice with PBS. Then CCK-8 solutions were incubated with MN9D cells for 1.5 h at 37°C, and the absorbance at 450 nm was measured via a microplate reader (PerkinElmer, Waltham, MA, USA). Culture medium containing CCK-8 solutions, in the absence of cells, was set as the blank control.

### Knockdown and overexpression in MN9D cells

Three small interfering RNAs (siRNAs) targeting CNTNAP4 sequences (siRNA-1, 5′- GCCTATAAGCACCAAGGAA-3′; siRNA-2, 5′-GCAGATACGATACAAGCTA-3′; siRNA-3, 5′-GCTCAATAGTCAACTCTTT-3′) were designed and synthesized by RioBio (Guangzhou, China), and negative-control siRNA was also provided by RioBio. Transfection was performed according to our previous work 23. Briefly, diluted siRNA (100 nM) was incubated with riboFECT CP Reagent (RioBio) for 15 min at room temperature (RT). Then, the mixture was added into the culture medium and incubated with MN9D cells. Cells were collected for Western blotting after 72 h of transfection. EGFP-vector and EGFP-CNTNAP4 plasmids were provided by Dongze Biotechnology Co., Ltd. (Guangzhou, China). MN9D cells were seeded at a density of 1.5 × 10^6^ cells/well at 50% confluency. Lipofectamine™ 3000 (Invitrogen) reagent or DNA was diluted in Opti-MEM™ medium and was then mixed. The mixture was incubated for 15 min at RT and was then added to cells. Two days after transfection, Western blotting and immunofluorescence were performed.

### RNA sequencing

At 72 h after transfection with CNTNAP4 siRNA, total RNA from MN9D cells was isolated using Trizol (Life Technologies, Carlsbad, CA, USA). Qubit® 2.0 Flurometer (Life Technologies, CA, USA) and Agilent 2200 TapeStation (Agilent Technologies, CA, USA) were used to measure RNA concentration and integrity. NEBNext Ultra RNA Library Prep Kit for Illumina (New England Biolabs [NEB], Ipswich, MA, USA) was used to prepare RNA libraries following the manufacturer's instructions, and an Agilent Bioanalyzer 2100 system was used to assess the quality of the purified library. Clustering of the index-coded samples was performed on a cBot Cluster Generation System using TruSeq PE Cluster Kit v3-cBot-HS (Illumina [NEB]) according to the manufacturer's instructions. After cluster generation, samples were sequenced on an Illumina HiSeq Xten platform (San Diego, CA, USA). All sequencing was performed at RiboBio Co., Ltd. (Guangzhou, China).

### Bioinformatic analysis

#### Analysis of differentially expressed genes

Quality control (QC) of RNA sequencing reads was performed using FastQC. Trimming was performed by seqtk for known Illumina TruSeq adapter sequences, poor reads, and ribosomal RNA reads. An index of the reference genome was built using STAR and paired-end clean reads were aligned to the reference genome using STAR (v2.5.1b). Then, HISAT2 was used to count the read numbers mapped to each gene, and fragments per kilobase of transcript per million mapped reads of each gene were calculated based on the length of the gene and its mapped read counts. Differential expression analysis of two groups was performed using the DESeq2 R package (1.10.1). The resulting *P*-values were adjusted using the Benjamini and Hochberg's approach for controlling the false discovery rate. Genes with an adjusted *p*-value < 0.05 and absolute values of log2(fold change) > 1 found by DESeq2 were assigned as being differentially expressed.

#### Functional enrichment analysis

Kyoto Encyclopedia of Genes and Genomes (KEGG) and Gene Ontology (GO) enrichment analysis of DEGs were performed via the R package (v 3.5.1).

### Immunocytochemical staining

Immunocytochemical staining was performed according to our previous work 22. MN9D cells or primary neurons grown in a confocal dish were incubated with primary antibodies overnight at 4°C, rinsed with PBS, and incubated with fluorescent-labeled secondary antibody for 1 h at 37°C. Then, 4',6-diamidino-2-phenylindole (DAPI) was used to stain cellular nuclei. The images were scanned under a confocal laser-scanning microscope (SP8; Leica, Hamburg, Germany).

To doubly stain LC3 with a mitochondrial tracer, 72 h after CNTNAP siRNA transfection, a LC3-GFP lentivirus and Mito-RFP lentivirus (provided by HanBio Co., Ltd., Shanghai, China) were co-transfected into MN9D cells for 24 h. After three washes with PBS, images were scanned under a confocal laser-scanning microscope (SP8; Leica).

### Stereotaxic injection of CNTNAP4 AAV-shRNA virus into the substantia nigra pars compacta

The CNTNAP4 AAV-shRNA virus was generated by ligating annealed oligonucleotides encoding a short-hairpin CNTNAP4 sequence into the *Bsp* EI/*Eco* RI site of the AAV2/9-TH-3Flag-miR30-GFP-polyA (pMT397) shRNA vector. CNTNAP4 AAV-shRNA was constructed to express shRNA targeting CNTNAP4 (GCTCAATAGTCAACTCTTT) via the TH promoter. CNTNAP4 AAV-shRNA and Ctrl AAV-shRNA were packaged by Sunbio Medical Biotechnology (Shanghai, China). CNTNAP4 AAV-shRNA (viral: 3.43 × 10^12^ particles mL^-1^) or Ctrl AAV-shRNA (viral: 2.85 × 10^12^ particles mL^-1^) were stereotaxically injected into the SN pars compacta (SNpc) as previously described 23. Mice were anesthetized and placed in a stereotaxic frame. CNTNAP4 AAV-shRNA or Ctrl AAV-shRNA in 0.5 µl of vol were delivered into the bilateral SNpc at the target site we reported previously (Bregma AP, -3.0 mm, ML, ±1.3 mm, DV, -4.7 mm) 23. The syringe was left in place for 5 min before being slowly withdrawn from the brain. To examine CNTNAP4 deficiency in DA neurons in the SNpc, mice were divided into the following four groups: Ctrl AAV-shRNA group, CNTNAP4 AAV-shRNA group, MPTP + Ctrl AAV-shRNA group, and MPTP + CNTNAP4 AAV-shRNA group. Three weeks after stereotaxic injections of the AAVs, mice in each group were subjected to chronic MPTP administration for another five weeks. Then, behavioral tests were performed and the mice in each group were sacrificed for the indicated experiments.

### Behavioral tests

#### Open field test

The open field test (OFT) was performed as previously described 24. Each mouse was placed into the center of the open field and was allowed to explore for 15 min under dim light. A video-tracking system, EthoVisione XT software (Beijing, China), was used to record the distance traveled as a measure of locomotor activity. The time spent in the center and entries into the center of the open field were also measured.

#### Motor coordination test

The motor coordination test was measured via the RotaRod for Mouse (Ugo basile SRL, Gemonio, VA, Italy). Mice were trained for three days before starting the motor coordination test. In the training phase, mice were placed on the rotarod so that they became familiarized with the revolving rotarod, which was set at 10 rpm for 5 min. Three days later, mice were subjected to the test phase. Mice were placed on the rotarod with an increasing speed (the initial speed was 4 rpm and the final speed was 40 rpm). The latency period was recorded as the time spent on the rotating bar.

#### Grip strength test

Neuromuscular strength testing was measure by using a grip strength meter (Ugo basile SRL, Gemonio, VA, Italy) as previously described 25. To assess grip strength, mice were allowed to grasp a metal grid with either of their fore limbs. The tail was gently pulled and the maximum holding force was recorded by the force transducer when the mice released their grasp on the grid. The peak holding strength was digitally recorded and displayed as force, measured in grams (g). Grip strength was also measured in g, and each mouse was assessed a total of three times in the grip strength test.

### Western blotting

Western blotting was performed as previously described 26. Samples were subjected to 10-12% sodium dodecyl sulfate polyacrylamide gel electrophoresis and were probed using the indicated primary antibodies. After being incubated with HRP-conjugated secondary antibodies, the protein strips were incubated with BeyoECL (Beyotime Biotechnology) and were illuminated via the GeneGnome XRQ Chemiluminescence imaging system (Gene Company, Hong Kong, China).

### Immunohistochemistry and immunofluorescent assays

Immunohistochemistry and immunofluorescence were performed as described previously 23, 27. Briefly, the embedded mouse brain was cut into 15-μm sections with a freezing microtome (Leica), and the slices were incubated with the corresponding primary antibodies overnight at 4°C. In the immunohistochemistry assay, the brain slices were incubated with a secondary antibody labeled with biotin for 1 h at RT. After three washes with PBS, the slices were stained with diaminobenzidine (DAB) and were scanned under a light microscope (Leica). For the immunofluorescent assay, fluorescent-labeled secondary antibodies were added and incubated for l h. After three washes with PBS for 5 min each, DAPI was added to stain nuclei for 3 min. Brain sections were scanned and imaged under a confocal laser-scanning microscope (SP8; Leica). Quantitative analysis was determined by the Image-Pro Plus 6.0 photogram analysis system (IPP 6.0, Media Cybernetics, Bethesda, MD, USA).

### Mitochondrial membrane potential

Measurement of the mitochondrial membrane potential (MMP) was performed according to the manufacturer's instructions. Briefly, after transfection with siRNA for 72 h, the culture medium was removed. MN9D cells were washed once with PBS and 1 mL of JC-1 staining solution was then added and mixed thoroughly. After incubation at 37°C for 20 min, the supernatant was discarded and washed two times with JC-1 staining buffer. Cells were then added to the culture medium, and images were scanned under a confocal laser-scanning microscope (SP8; Leica).

### Transmission electron microscopy

Ultrastructural morphology of the SN and MN9D cells were analyzed by transmission electron microscopy (TEM). Mice were anaesthetized and the SN in the midbrain was dissected and fixed with TEM fixative solution at 4°C for 2-4 h. MN9D cells were removed from the culture medium and were fixed with TEM fixative solution at 4°C for 2-4 h. After three washes with 0.1 M of phosphate buffer (pH 7.4), the SN and MN9D cells were fixed with 0.1 M of phosphate buffer (pH 7.4) with 1% osmium at RT for 2 h. Samples were then dehydrated by sequential incubations in 50%, 70%, 80%, 90%, 95%, and 100% ethanol, followed by incubation in 100% acetone. Tissues and cells were permeated with acetone and 812 embedding agents for 2-4 h and were then incubated in an 812 embedding oven (SPI-Pon 812 Epoxy Resin Monomer; SPI, Shanxi, China) overnight at 37°C. The samples were sliced into ultra-thin sections of 60-80 nm with an ultra-thin slicer (Leica); the slices were double stained with uranium and lead at RT. Autolysosomes, autophagosomes, and synaptic vesicles were observed under a transmission electron microscope (HT7700; Hitachi, Tokyo, Japan), and images were collected for analysis according to the methods described by Moss and Bolam, with minor modifications 28.

### Patient characteristics

Plasma samples were collected from 90 PD patients (mean age 64.13 ± 11.25 years, 54 male, 36 female) and 90 controls (mean age 63.18 ± 15.43 years, 40 male, 50 female). CSF samples were enrolled and collected as previously described in our recent study^29^, and included 58 patients with PD (56.86 ± 9.43 years, 31 males, 27 females) and 21 healthy controls (53.90 ± 19.27 years, 12 males, 9 females). All subjects were enrolled from the First Affiliated Hospital of Guangzhou Medical University. PD patients were diagnosed using the clinical criteria from the UK Parkinson's Disease Society Brain Bank 30. The procedures were approved by the Ethics Committee of the First Affiliated Hospital of Guangzhou Medical University, and all patients and controls provided written informed consent. Age- and gender-matched controls were identified during routine health examinations. CSF and plasma levels of CNTNAP4 were measured as described below.

### Enzyme-linked immunosorbent assay

Human CSF and plasma CNTNAP4 concentrations were measured with enzyme-linked immunosorbent assay (ELISA) kits (AndyGene Biotechnology Co., Ltd., Beijing, China). Human plasma was collected as stated above, and ELISAs were performed according to the manufacturer's instructions. Briefly, 20 µL of samples were mixed with sample solution and were then pipetted into 96-well plates pre-coated with an antibody specifically against human CNTNAP4. After incubation at 37°C for 30 min, the plates were washed five times, and 50 µL HRP-conjugated secondary antibody was added. After incubation at 37°C for 30 min, the substrate solution (50 µL chromogen solution A and 50 µL chromogen solution B) was added to each well for another incubation at 37°C for 15 min. Stop solution was used to terminate the reaction. The absorbance at 450 nm was measured with a microplate reader (PerkinElmer). Data were obtained from three separate experiments, each of which were performed in triplicate.

### Statistics

Statistical tests were performed using GraphPad Prism 8.0 (GraphPad Software, La Jolla, CA) via one-way or two-way analysis of variance (ANOVA) followed by Tukey's *post-hoc* tests for multiple comparisons, whereas the Student's *t*-test was used for comparisons between only two groups. All data are expressed as the mean ± standard error of the mean (SEM), and the statistical significance level was set at *p* < 0.05.

## Results

### CNTNAP4 expression is decreased in MPTP mouse models of PD

**Figure 1:**
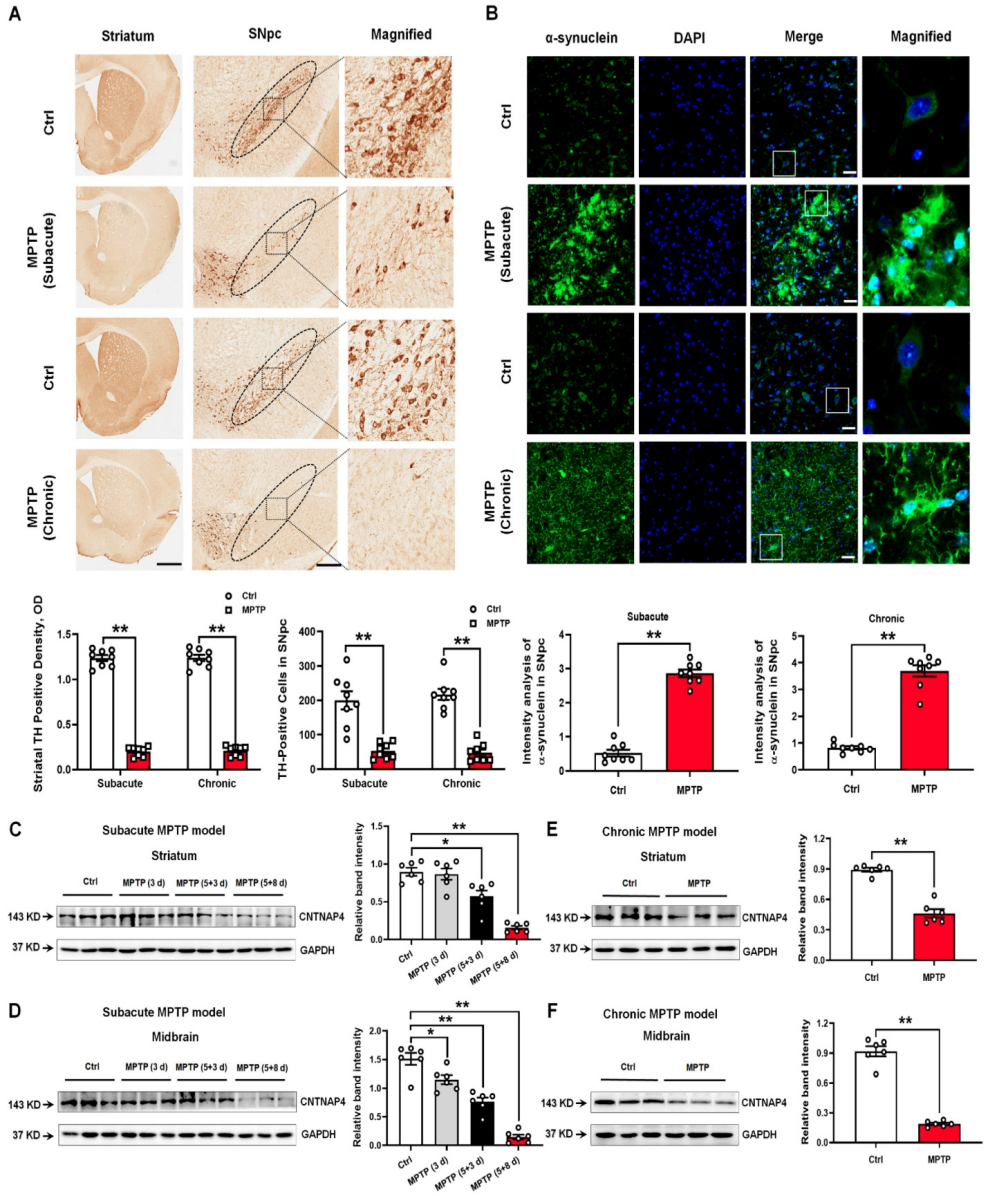
** Expression patterns of TH, α-synuclein, and CNTNAP in the SNpc and striatum of subacute and chronic MPTP mouse models.** (A) Immunohistochemical staining of TH-positive cells in the striatum and SNpc in subacute and chronic MPTP mouse models. Magnified TH-positive cells in the SNpc are shown in the right column of panel A (scale bars, 1 mm in the striatum and 100 µm in the SNpc). The ellipses in the middle column of panel A denote the boundaries of the SNpc and the middle-column boxes denote the areas that are expanded in the right-hand columns in panel A. Quantification of striatal TH-positive density and TH-positive cells in the SNpc was shown as below panel A. (B) Immunofluorescent staining of α-synuclein-positive cells in the SNpc in subacute and chronic MPTP mouse models. Magnified α-synuclein-positive cells in the SNpc are shown in the right column of panel B (scale bars, 25 µm). Quantification of α-synuclein expression in the SNpc is shown at the bottom of panel B. (C and D) CNTNAP4 expression levels in the striatum and midbrain at different time points upon subacute MPTP administration (3 d, 5+3 d, and 5+8 d), as described in the Materials and Methods, was determined by Western blotting (n = 6 per group). (E and F) CNTNAP4 expression in the striatum and midbrain in the chronic MPTP model was determined by Western blotting (n = 6 per group). Results are expressed as the mean ± SEM. ^**^p < 0.01, ^*^p < 0.05 vs. control group. Statistical significance was determined by Student's *t*-tests for A, B, E and F, and by one-way ANOVAs for C and D.

To obtain the general expression pattern of CNTNAP4 in PD animal models, we generated subacute and chronic MPTP mouse models. We found that TH expression was significantly decreased and α-synuclein expression was significantly increased in the striatum and SNpc in both subacute and chronic MPTP models (Figure 1A-B). These results suggested that our subacute and chronic MPTP models successfully mimicked pathological phenotypes of parkinsonism. Then we found that CNTNAP4 expression was significantly decreased in the striatum and midbrain in the subacute and chronic MPTP model (Figure 1C-F). We also confirmed these results by co-staining CNTNAP4 and α-synuclein in the SNpc in the chronic MPTP model and found that MPTP induced a differential expression pattern of CNTNAP4 and α-synuclein in the SNpc (Figure S1). These results suggest that decreased CNTNAP4 expression may be correlated with nigrostriatal DA neuronal loss and augment α-synuclein expression in the MPTP model.

### CNTNAP4 knockdown increases α-synuclein and decreases synapsin levels in DA MN9D cells *in vitro*, whereas CNTNAP4 overexpression induces an opposite effect on these expression levels

**Figure 2:**
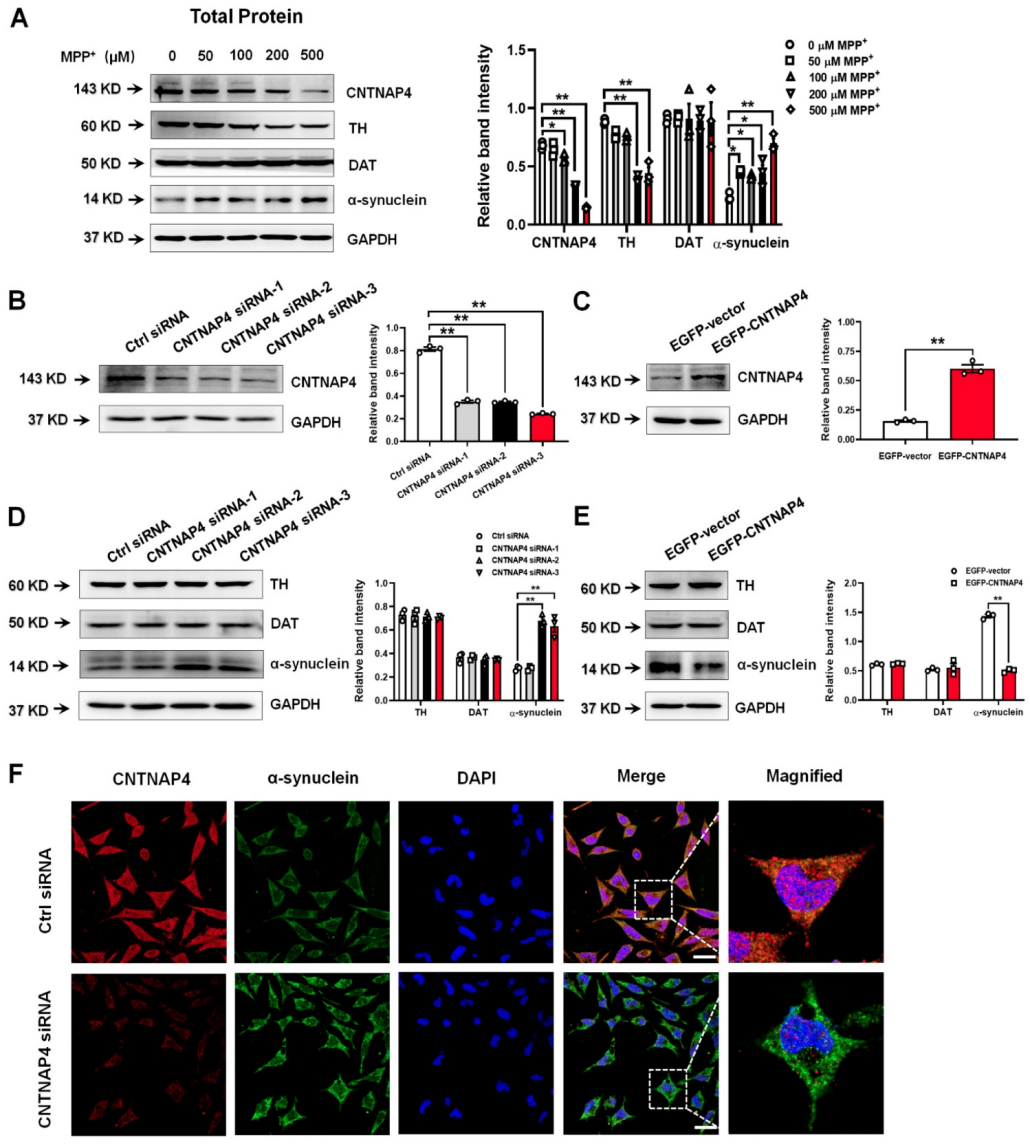
** CNTNAP4 knockdown increases α-synuclein expression in MN9D cells.** (A) Effects of different concentrations of MPP^+^ on CNTNAP4, TH, DAT, and α-synuclein expression levels in MN9D cells were determined by Western blotting. (B and C) The interference or overexpression efficiency of transfected CNTNAP4 siRNAs or plasmids in MN9D cells were confirmed by Western blotting. (D and E) Effects of CNTNAP4 siRNA or plasmid transfection on TH, DAT, and α-synuclein expression levels in MN9D cells were determined by Western blotting. (F) Immunocytochemical staining of α-synuclein upon CNTNAP4 knockdown in MN9D cells. Magnified CNTNAP4 and α-synuclein staining are shown in the right-hand column, which are expansions of the boxed areas in the merged column (scale bars, 20 µm; n = 3 per group). Results are expressed as the mean ± SEM. ^**^p < 0.01, ^*^p < 0.05 vs. control group. Statistical significance was determined by one-way ANOVAs for A, B and D, and Student's *t*-tests for C and E.

Since CNTNAP4 is highly enriched in DA neurons 11, 18 and we found that CNTNAP4 expression was strongly correlated with parkinsonian phenotypes, we next investigated the role of CNTNAP4 in DA neurons *in vitro*. Here, we firstly used MPP^+^ to treat MN9D cells, a well-established DA precursor cell line, and measured MN9D cell viability following exposure to different concentrations of MPP^+^ (Figure S2A). Consistently, we found that MPP^+^ decreased TH and CNTNAP4 expression levels and increased α-synuclein expression in MN9D cells, whereas MPP^+^ did not change the expression of dopamine transporter (DAT; Figure 2A). Next, we generated three siRNA sequences targeting CNTNAP4, and we found that the third sequence possessed the most significant knockdown efficiency (Figure 2B). In addition, we also successfully generated a CNTNAP4 plasmid to overexpress CNTNAP4 in MN9D cells (Figure 2C). Subsequently, we found that CNTNAP4 knockdown increased—while CNTNAP4 overexpression decreased—α-synuclein expression in MN9D cells (Figure 2D-F). However, we did not find a direct interaction between α-synuclein and CNTNAP4 using immunoprecipitation (Figure S2B). Thus, the above results suggest that CNTNAP4 may have indirectly changed α-synuclein expression patterns in DA neurons rather than via a direct interaction.

**Figure 3:**
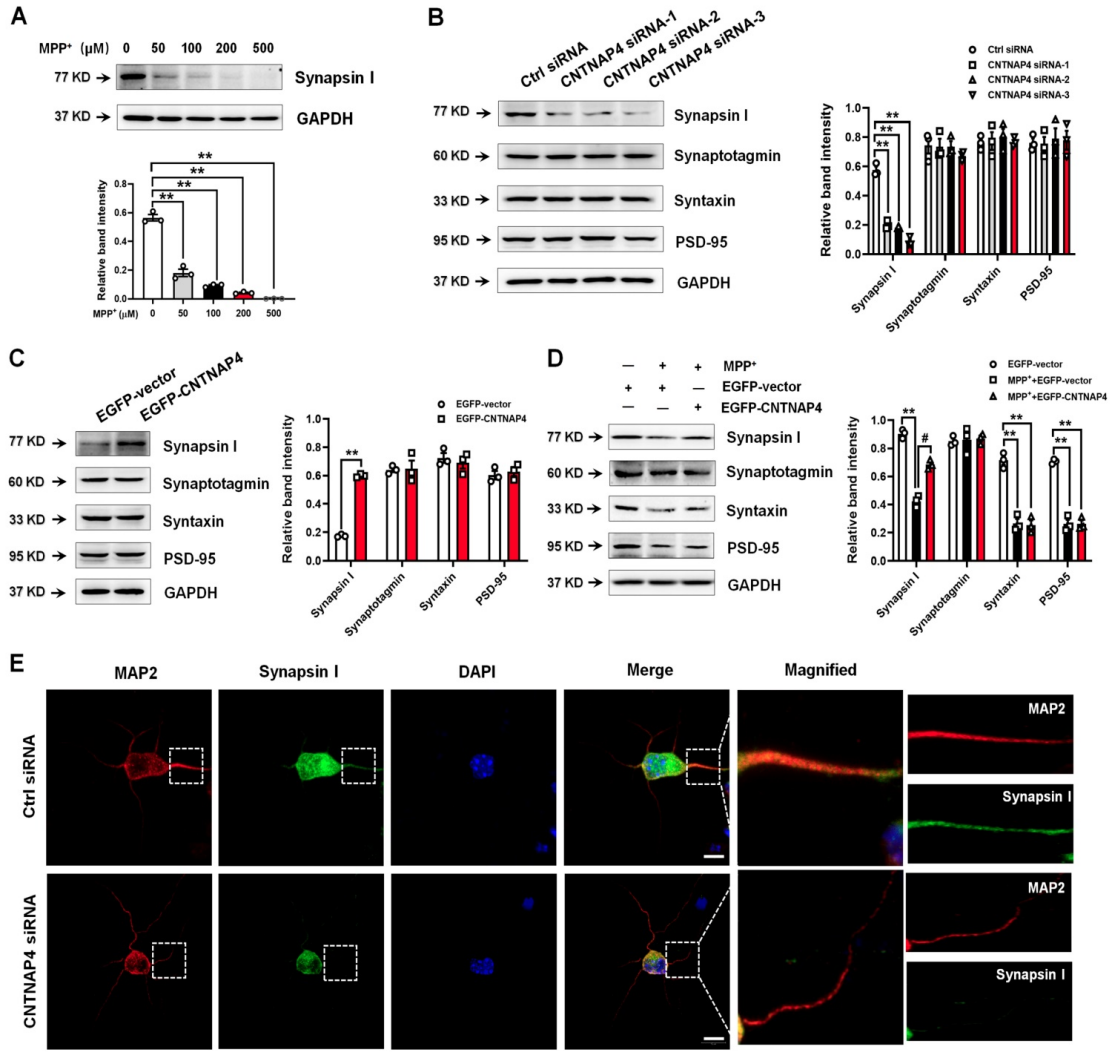
** CNTNAP4 overexpression rescues MPP^+^-induced decreases in synapsin-І expression in MN9D cells.** (A) Effects of different concentrations of MPP^+^ on synapsin-І expression in MN9D cells were determined by Western blotting. (B and C) Effects of CNTNAP4 siRNA or overexpression on expression levels of synapsin І, synaptotagmin, syntaxin, and PSD-95 in MN9D cells were determined by Western blotting. (D) Effects of CNTNAP4 plasmid transfection on expression levels of synapsin І, synaptotagmin, syntaxin and PSD-95 in MPP^+^-treated MN9D cells were determined by Western blotting. (E) Immunocytochemical staining of MAP-2 and synapsin І upon CNTNAP4 knockdown in MN9D cells. Magnified MAP-2 and synapsin-І staining are shown in the right-hand column, which are expansions of the boxed areas in the merged column (scale bars, 20 µm; n = 3 per group). ^**^p < 0.01 vs. control group; ^#^*p* < 0.05 vs. MPP^+^ group. Statistical significance was determined by one-way ANOVAs for A, B and D, and a Student's *t*-test for C.

Because CNTNAP4 plays an important role in synaptic function, we next investigated whether CNTNAP4 has an impact on synaptic proteins. We found that MPP^+^ above 100 µM significantly decreased both pre- and post-synaptic proteins, such as synapsin І, syntaxin, synaptosome associated protein 25 (SNAP25), and postsynaptic density 95 (PSD-95; Figure 3A and Figure S2C). Among these synaptic proteins, synapsin І exhibited the most sensitive response to MPP^+^, as 50 µM of MPP^+^ significantly abolished its expression (Figure 3A). In addition, we found that CNTNAP4 knockdown decreased while CNTNAP4 overexpression increased synapsin І expression in MN9D cells, without obvious changes of synaptotagmin, syntaxin, or PSD-95 (Figure 3B-C). Importantly, we found that CNTNAP4 plasmid rescued expression of synapsin І compared with that of an EGFP vector in MPP^+^-treated MN9D cells, whereas the CNTNAP4 plasmid did not change the expression levels of synaptotagmin, syntaxin, or PSD-95 (Figure 3D). We further confirmed our results in primary neuronal cultures, as we found that CNTNAP4 knockdown decreased synapsin І expression, especially in the neurites of primary neurons (Figure 3E). These results suggest that CNTNAP4 increases the expression of synapsin І in DA MN9D cells.

### Transcriptomic landscape following CNTNAP4 knockdown in DA MN9D cells

**Figure 4:**
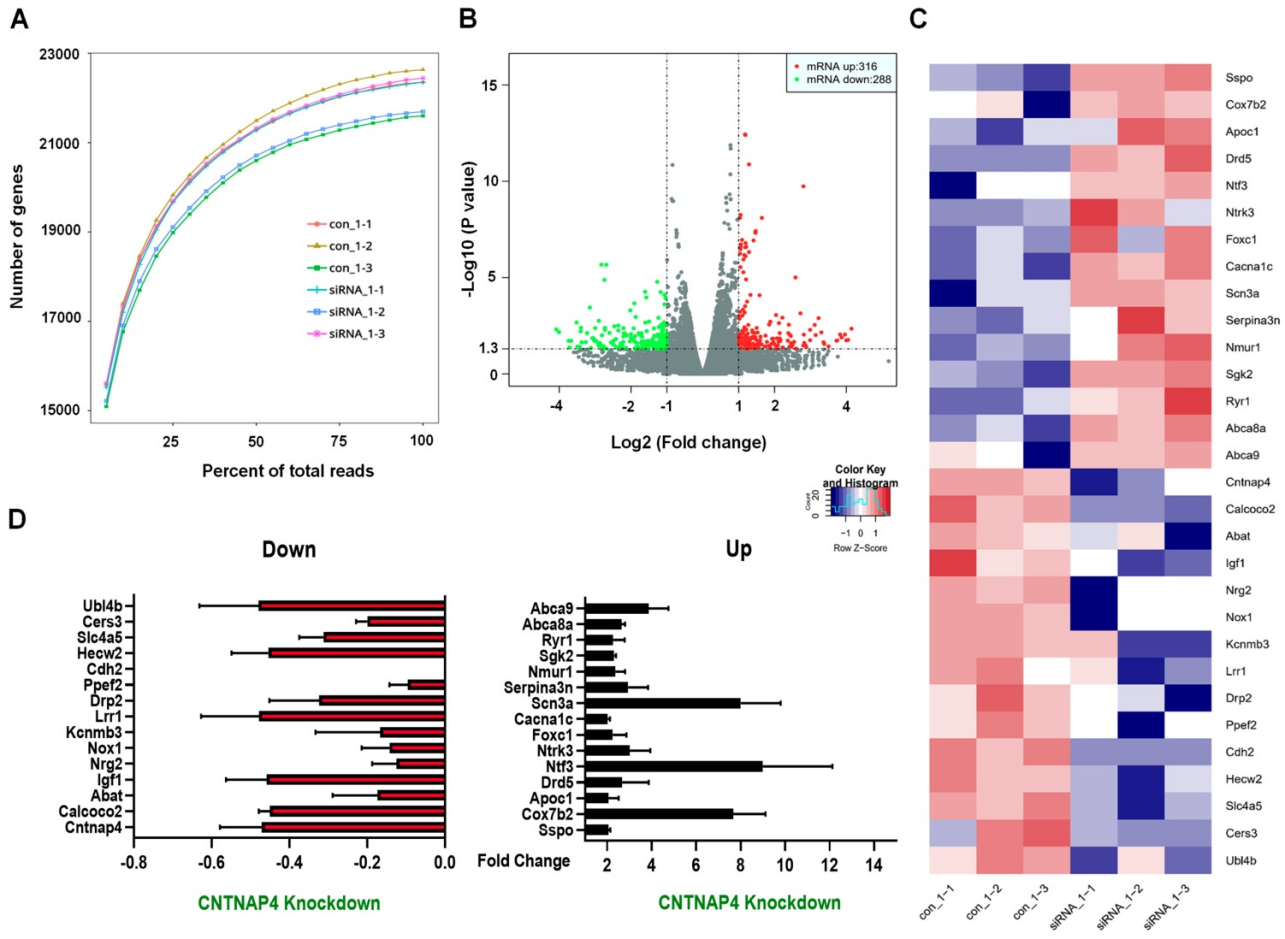
** Transcriptomic profiles following CNTNAP4 knockdown in MN9D cells.** (A) Sequencing saturation results for Ctrl group and CNTNAP4 siRNA group are shown. (B) DEGs are shown as a volcano plot. (C) Heatmap of DEGs in Ctrl group and CNTNAP4 siRNA group. (D) Upregulated and downregulated DEGs upon CNTNAP4 knockdown in MN9D cells are shown. Note that CNTNAP4 knockdown decreased CNTNAP4 and calcoco2 expression levels in MN9D cells. Genes with an adjusted *p* < 0.05 and absolute values of log2(Fold Change) > 1 found by DESeq2 were assigned as being differentially expressed.

We then performed RNA-seq to determine the transcriptomic landscape following CNTNAP4 knockdown in MN9D cells. Sequencing saturation results for each group are shown in Figure 4A. There were 604 DEGs (316 upregulated and 288 downregulated) across different chromosomes, and a volcano map of these DEGs is shown in Figure 4B. Subsequently, we performed KEGG and GO analysis to reveal the potential pathways involved in these DEGs. Functional analysis showed that 46 DEGs were enriched in molecular functions related to catalytic activity, 63 DEGs were enriched in membrane-related cellular components, 62 DEGs were enriched in biological processes related to responses to stimuli, and 28 DEGs were enriched in biological processes related to cellular proliferation (Figure S3). Furthermore, KEGG analysis revealed that five DEGs (*Tacr2, Drd5, Nmur1, Calcrl,* and* Avpr2*) were enriched in neuroactive ligand-receptor interactions, and that three DEGs (*Cldn3, Tigit,* and* Cdh2*) were enriched in cell-adhesion molecules (CAMs; Figure S4). Figure 4C-D provides a list of the hierarchical clustering of 30 DEGs related to synaptic function in MN9D cells upon CNTNAP4 knockdown. Additionally, RNA-seq results also confirmed our CNTNAP4 siRNA knockdown efficiency in MN9D cells (Figure 4C-D). Thus, our RNA-seq data reveal that CNTNAP4 knockdown in MN9D cells induces changes in the expression levels of many proteins that are associated with synaptic function.

### CNTNAP4 knockdown induces mitophagy in DA MN9D cells

**Figure 5:**
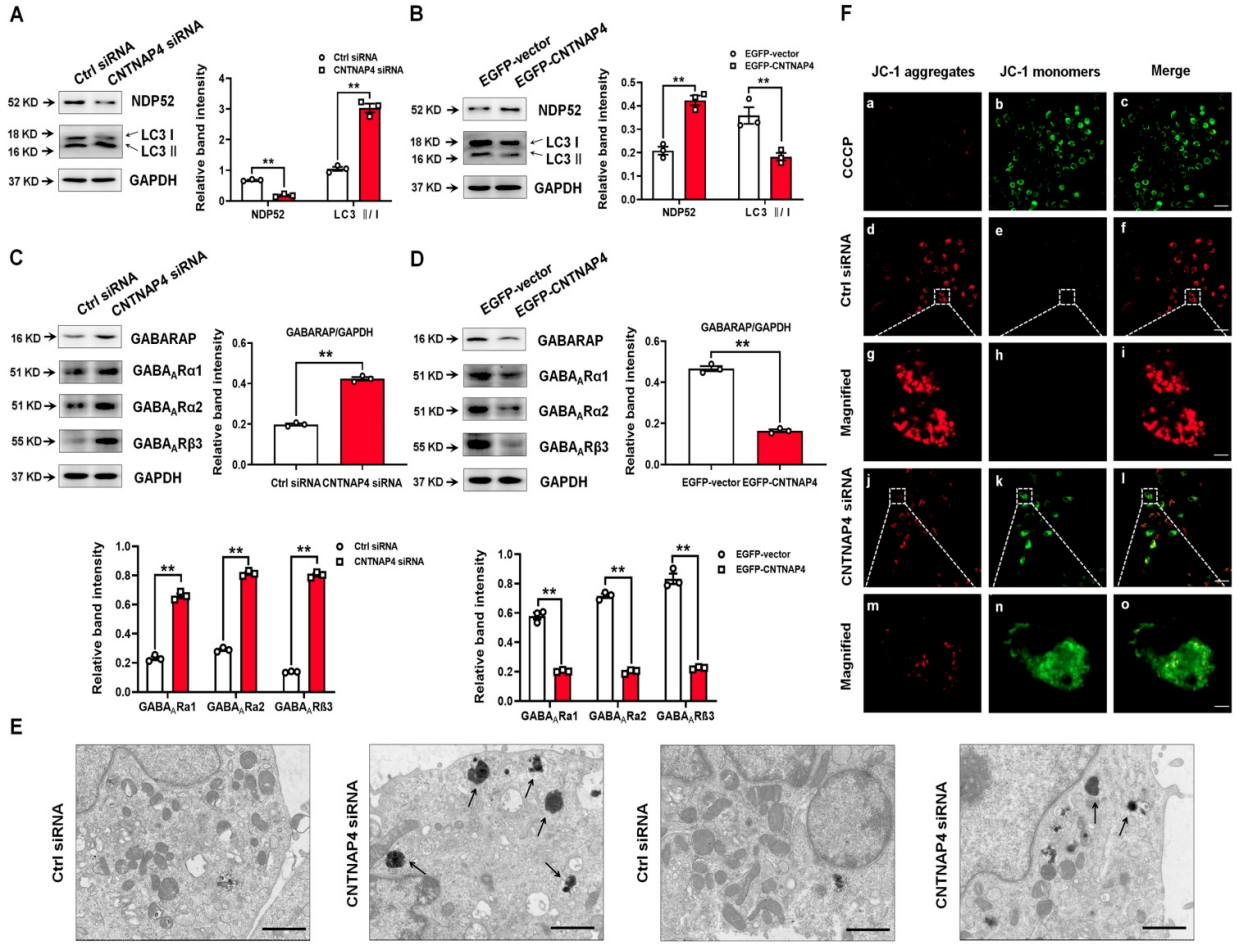
** CNTNAP4 knockdown induces autophagy and impairs mitochondrial function in MN9D cells.** (A and B) Effects of CNTNAP4 siRNA or plasmid transfection on the expression levels of NDP52 and LC3 in MN9D cells were determined by Western blotting. (C and D) Effects of CNTNAP4 siRNA or plasmid transfection on the expression levels of GABARAP, GABA_A_Rα1, GABA_A_Rα2, and GABA_A_Rβ3 in MN9D cells were determined by Western blotting. (E) Loss of CNTNAP4 results in increased autolysosomes in MN9D cells (as indicated by the black arrows; scale bars, 1 µm). (F) Effect of CNTNAP4 knockdown on the MMP using JC-1 fluorescent probes in MN9D cells (scale bars, 30 µm in a-f and j-l; 3 µm in g-i and m-o; n = 3 per group). Magnified images are expansions of the boxed areas in the corresponding panels above the “Magnified” columns. Results are expressed as the mean ± SEM. ^**^p < 0.01 vs. control group. Statistical significance was determined by Student's *t*-tests.

Our RNA sequencing data showed that calcium binding and coiled-coil domain 2 (*calcoco2*, which encodes NDP52) was decreased by 55% upon MN9D knockdown (Figure 4D). Regarding its protein level, CNTNAP4 knockdown decreased—whereas CNTNAP4 overexpression increased—NDP52 expression in MN9D cells (Figure 5A-B). Because NDP52 has previously been reported to be a key regulator for selective autophagy, and LC3/GABARAPs, which are autophagy-related 8 (Atg8) homologs, amplify mitophagy by recruitment of NDP52 31, 32. We also examined the expression of LC3 and GABARAP in MN9D cells. CNTNAP4 knockdown increased—whereas CNTNAP4 overexpression decreased—the LC3-II/LC3-І ratio and GABARAP expression (Figure 5A-D). Furthermore, CNTNAP4 knockdown increased while CNTNAP4 overexpression decreased GABA_A_-type receptors, such as GABA_A_Rα1, GABA_A_Rα2, and GABA_A_Rβ3 (Figure 5C-D). These results suggest that CNTNAP4 deficiency may induce autophagy in MN9D cells.

Then we used TEM to visually examine autophagy in MN9D cells following CNTNAP4 knockdown. We found that CNTNAP4 knockdown reduced the number of mitochondria and increased the formation of autolysosomes in MN9D cells (Figure 5E). To further examine the effect of CNTNAP4 knockdown on mitochondrial function, we evaluated the MMP using JC-1 fluorescent probes. In the normal condition, JC-1 aggregated within the mitochondrial matrix, and formed J-aggregates (Figure 5F). When the MMP was reduced, JC-1 aggregates turned into free monomers. Here, we found that CNTNAP4 knockdown induced JC-1 aggregates turning into monomers (Figure 5F), suggesting that CNTNAP4 knockdown decreased the MMP and disrupted mitochondrial function in MN9D cells.

**Figure 6:**
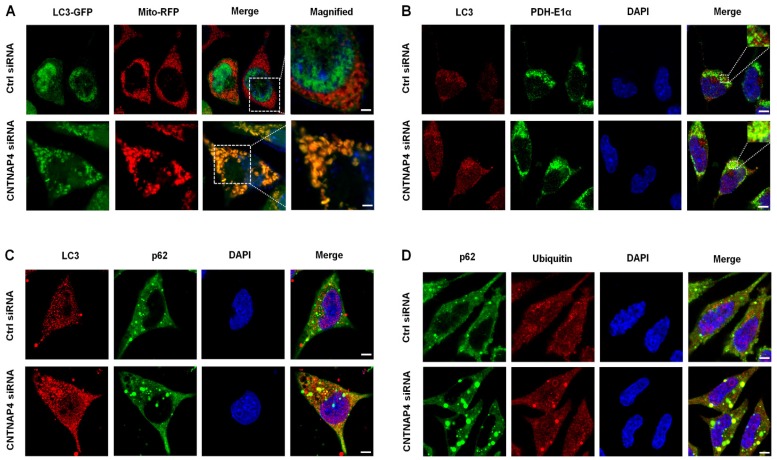
** CNTNAP4 knockdown induces mitophagy in MN9D cells.** (A) After transfection with CNTNAP4 siRNA for 72 h, MN9D cells were then transfected with an LC3-GFP lentivirus and a Mito-RFP lentivirus. The images show the interaction between LC3 and the mitochondrial tracer, Mito. Magnified images are expansions of the boxed areas in the “Merge” column. (B-D) Immunocytochemical staining of LC3 and PDH-E1α, LC3 and p62, and p62 and ubiquitin following CNTNAP4 knockdown in MN9D cells (scale bars, 1.5 µm in A and 3 µm in B-D).

We generated an LC3-GFP lentivirus and a Mito-RFP lentivirus to determine whether CNTNAP4 knockdown induced mitophagy in MN9D cells. We found that LC3-GFP was co-expressed with Mito-RFP upon CNTNAP4 knockdown (Figure 6A), suggesting that CNTNAP4 knockdown induced mitophagy. We further confirmed our results via double immunostaining using LC3 and PDH-E1α, a specific mitochondrial marker, that revealed that CNTNAP4 knockdown increased the interaction between LC3 and PDH-E1α in MN9D cells (Figure 6B). CNTNAP4 knockdown also induced an interaction between LC3 and p62, suggesting that CNTNAP4 knockdown may induce mitophagy (Figure 6C). Additionally, impairment of autophagy is accompanied by accumulation of p62, which leads to the formation of large aggregates, including p62 and ubiquitin 33. We found an accumulation of p62 and ubiquitin in MN9D cells upon CNTNAP4 knockdown (Figure 6D). These results suggest that CNTNAP4 deficiency disrupts mitochondrial function and induces mitophagy to clear damaged mitochondria and nearby damaged proteins.

### CNTNAP4 deficiency in SNpc DA neurons induces parkinsonian phenotypes *in vivo*

**Figure 7:**
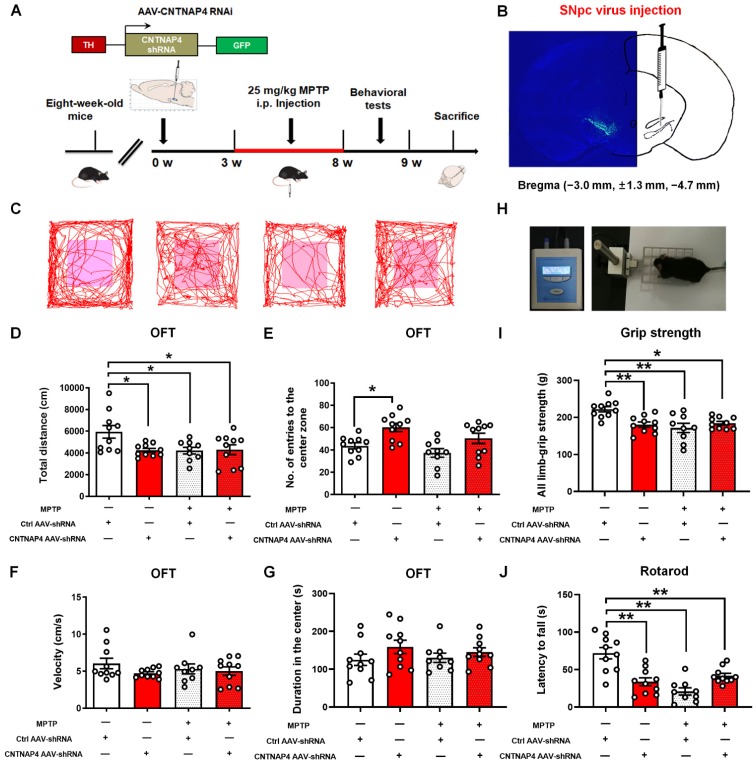
** CNTNAP4 knockdown in SNpc DA neurons induces motor dysfunctions in mice.** (A) The experimental timeline is shown. Three weeks after the CNTNAP4 AAV-shRNA or control AAV-shRNA was stereotaxically injected into the SNpc, mice were intraperitoneally administrated saline or MPTP for five weeks. One day after the last MPTP/saline injection, behavioral tests were performed, after which the mice were sacrificed. (B) AAV2/9-TH-3Flag-miR30-GFP-CNTNAP4 was bilaterally injected into the SNpc of mice. Representative image of CNTNAP4 AAV-shRNA-labeled neurons in the SNpc. (C) Representative path tracings in the OFT. (D-G) Total distance travelled, number of entries to the center zone, movement speed, and time spent in the center of the open-field in control, CNTNAP4 AAV-shRNA, MPTP, and MPTP+CNTNAP4 siRNA groups. (H) Representative image extracted from a mouse in the grip strength test. (I) The grip strength test was used to examine the limb grip strength of mice. (J) The rotarod test was used to examine the motor coordination of mice (n = 10 for each of the control, CNTNAP4 AAV-shRNA, and MPTP+CNTNAP4 siRNA groups; n = 9 in the MPTP group). Results are expressed as the mean ± SEM. ^**^p < 0.01, ^*^p < 0.05 vs. Control group. Statistical significance was determined by one-way ANOVAs and Tukey tests for *post-hoc* comparisons.

Next, we generated AAVs targeting the third CNTNAP4 siRNA sequence with a TH promoter (Figure S5). We delivered AAV-TH-Ctrl shRNA and AAV-TH-CNTNAP4 shRNA to the bilateral SNpc and, three weeks later, administrated MPTP or saline for another five weeks (Figure 7A). We found strong localization of CNTNAP4 AAV-shRNA in the SNpc region (Figure 7B). First, we performed behavioral tests to examine the effects of CNTNAP4 knockdown on motor function. We found that the total distance traveled was decreased in the CNTNAP4 AAV-shRNA, MPTP, and MPTP + CNTNAP4 AAV-shRNA groups as compared with that of the control group (Figure 7C-D). Although CNTNAP4 AAV-shRNA increased the number of entries to the center zone of the open field compared with that if the control group, there were no differences in terms of movement speed or the duration of time spent in the center zone in the open field among these four groups (Figure 7E-G). Furthermore, CNTNAP4 AAV-shRNA, as well as MPTP and MPTP + CNTNAP4 AAV-shRNA, decreased the limb-grip strength and latency to fall in mice compared with those of the control group in the grip strength and rotarod tests (Figure 7H-J). Thus, these results demonstrate that CNTNAP4 knockdown in SNpc DA neurons induced motor dysfunctions and that these effects were equivalent to MPTP administration in mice.

**Figure 8:**
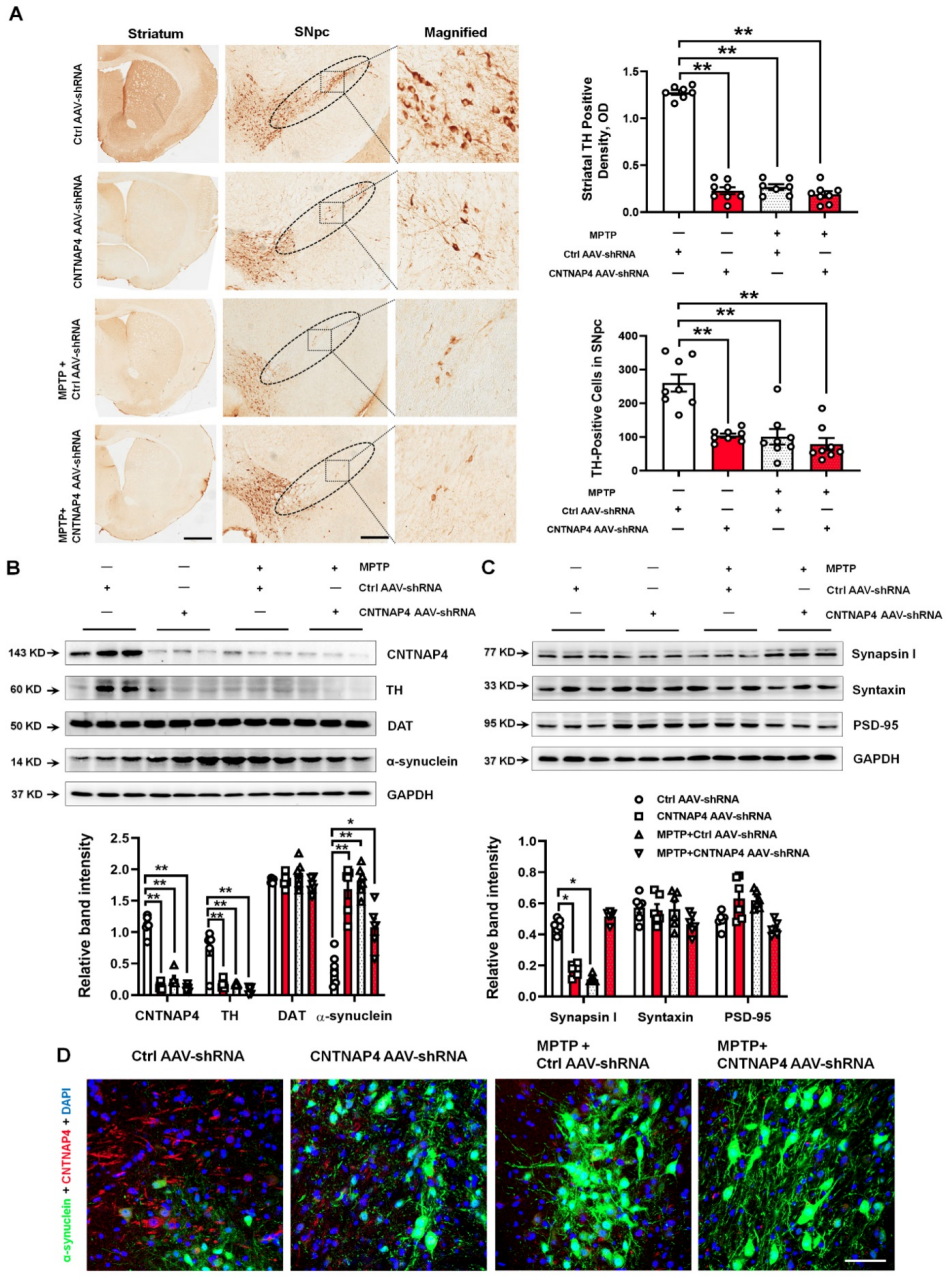
** CNTNAP4 knockdown in SNpc DA neurons induces DA neuronal degeneration and increased α-synuclein expression in mice.** (A) Immunohistochemical staining of TH-positive cells in the striatum and SNpc in control, CNTNAP4 AAV-shRNA, MPTP, and MPTP+CNTNAP4 siRNA groups (scale bars, 1 mm in striatum and 100 µm in SNpc). The ellipses in the middle column of panel A denote the boundaries of the SNpc and the middle-column boxes denote the areas that are expanded in the right-hand columns in panel A. Quantification of striatal TH-positive density and TH-positive cells in the SNpc was shown in the right of panel A. (B and C) Expression levels of CNTNAP4, TH, DAT, α-synuclein, synapsin І, syntaxin, and PSD-95 in the midbrain of control, CNTNAP4 AAV-shRNA, MPTP, and MPTP+CNTNAP4 siRNA groups were determined by Western blotting. (D) Immunofluorescent staining of CNTNAP4 and α-synuclein in the SNpc of Control, CNTNAP4 AAV-shRNA, MPTP, and MPTP+CNTNAP4 siRNA groups (scale bar, 25 µm). Western blotting results are from three of the six mice in each group and are expressed as the mean ± SEM. ^**^p < 0.01, ^*^p < 0.05 vs. Control group. Statistical significance was determined by one-way ANOVAs and Tukey tests for *post-hoc* comparisons.

Next, CNTNAP4 AAV-shRNA, as well as MPTP and MPTP + CNTNAP4 AAV-shRNA, significantly decreased TH expression in the striatum and SNpc compared with that of the control group (Figure 8A-B). We also confirmed our CNTNAP4 AAV-shRNA knockdown deficiency in the midbrain (Figure 8B). Moreover, CNTNAP4 AAV-shRNA, as well as MPTP and MPTP + CNTNAP4 AAV-shRNA, significantly increased α-synuclein expression in the midbrain as compared with that of the control group (Figure 8B). In addition, neither CNTNAP4 AAV-shRNA nor MPTP affected DAT expression (Figure 8B). Regarding expression levels of synaptic proteins, CNTNAP4 AAV-shRNA or MPTP decreased the expression of synapsin І in the midbrain as compared with that of the control group (Figure 8C), which was consistent with our previous *in vitro* results. However, MPTP + CNTNAP4 AAV-shRNA slightly increased the expression of synapsin І expression as compared with that of the control group (Figure 8C), which may have been due to compensatory effects. We also confirmed, via immunofluorescence, that CNTNAP4 AAV-shRNA increased α-synuclein expression in the SNpc (Figure 8D). These results suggest that CNTNAP4 knockdown in SNpc DA neurons induces parkinsonian phenotypes.

### Loss of CNTNAP4 results in ultrastructural deficits at DA synapses in the SNpc

**Figure 9:**
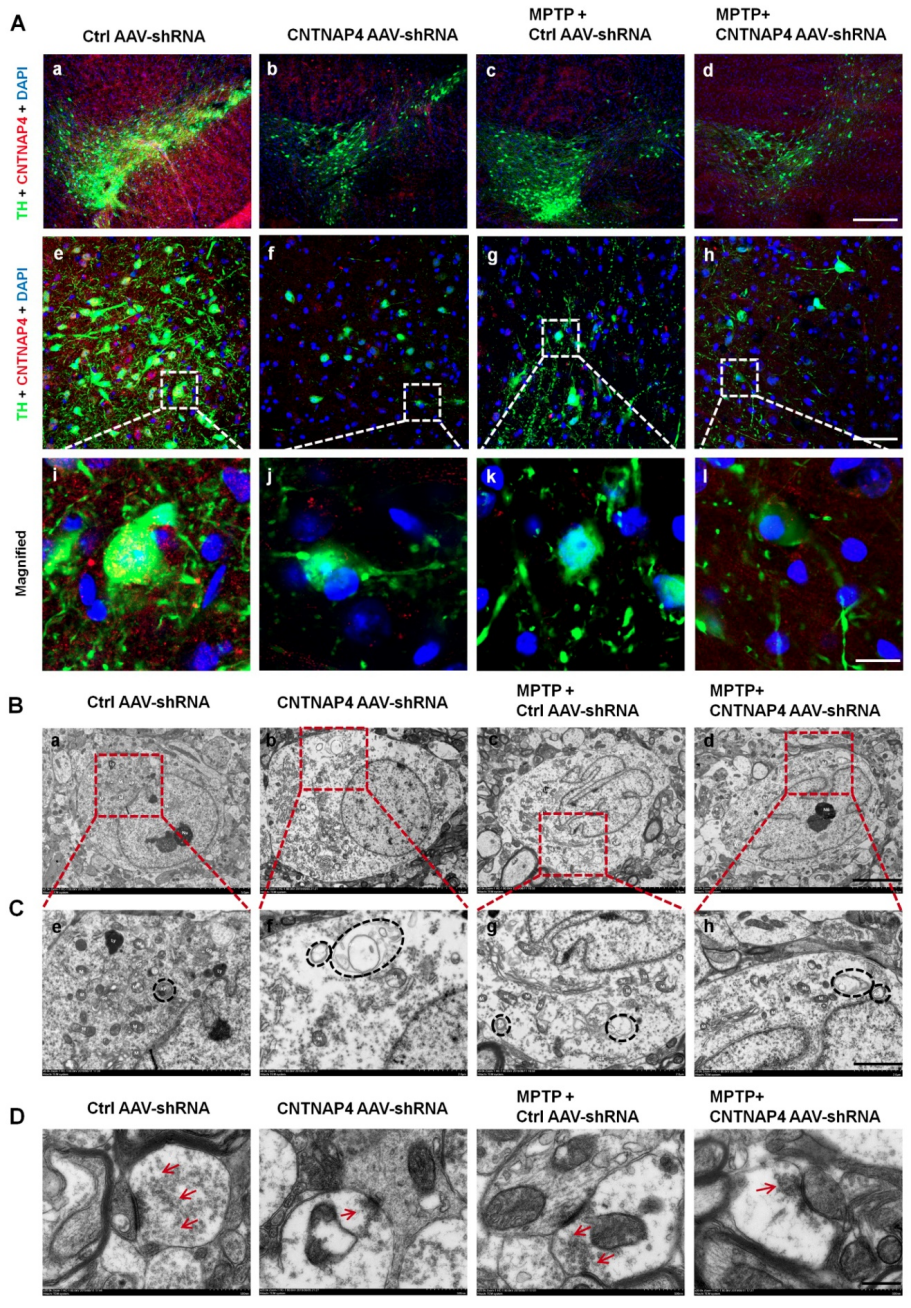
** CNTNAP4 knockdown in SNpc DA neurons induces ultrastructural deficits at DA synapses in the SNpc in mice.** (A) Immunofluorescent staining of CNTNAP4 and TH in the SNpc of control, CNTNAP4 AAV-shRNA, MPTP, and MPTP+CNTNAP4 siRNA groups. Magnified images are expansions of boxed areas in corresponding panels above each magnified image. (B and C) Ultrastructural analysis of autophagosomes in the SNpc of control, CNTNAP4 AAV-shRNA, MPTP, and MPTP+CNTNAP4 siRNA groups. Magnified images in C are expansions of corresponding panels above in panel B. (D) Ultrastructural analysis of synaptic vesicles in SNpc of control, CNTNAP4 AAV-shRNA, MPTP, and MPTP+CNTNAP4 siRNA groups. Quantification of synaptic vesicles in panel D are shown in **Figure S6**. Scale bars for A, 125 µm in a-d, 25 µm in e-h, and 5 µm in i-l; scale bar for B, 5 µm; scale bar for C, 2 µm; and scale bar for D, 500 nm.

Consistently, we also found that CNTNAP4 AAV-shRNA, as well as MPTP and MPTP + CNTNAP4 AAV-shRNA, significantly decreased TH and CNTNAP4 expression in SNpc DA neurons (Figure 9A). Since we found that CNTNAP4 knockdown induced a reduction in DA neurons and the level of synapsin І both *in vivo* and *in vitro*, we next explored the effects of CNTNAP4 knockdown on the ultrastructure of afferents innervating DA somata in the SNpc.

Consistent with the mitophagy induced by CNTNAP4 knockdown *in vitro*, we found that CNTNAP4 AAV-shRNA also reduced mitochondrial number and increased autophagosomes in the cytoplasm of DA neurons in the SNpc (Figure 9B-C). MPTP and MPTP + CNTNAP4 AAV-shRNA exhibited similar effects as those of CNTNAP4 AAV-shRNA in the SNpc (Figure 9B-C). The TEM results also revealed that the number of synaptic vesicles was decreased in CNTNAP4 AAV-shRNA, MPTP, and MPTP + CNTNAP4 AAV-shRNA groups as compared with that in the control group (Figure 9D and quantitation of synaptic vesicles is shown in Figure S6). These results suggest that CNTNAP4 knockdown induces ultrastructural deficits at DA synapses.

### CNTNAP4 knockout mice show movement deficits and reduced TH expression in the SNpc

**Figure 10:**
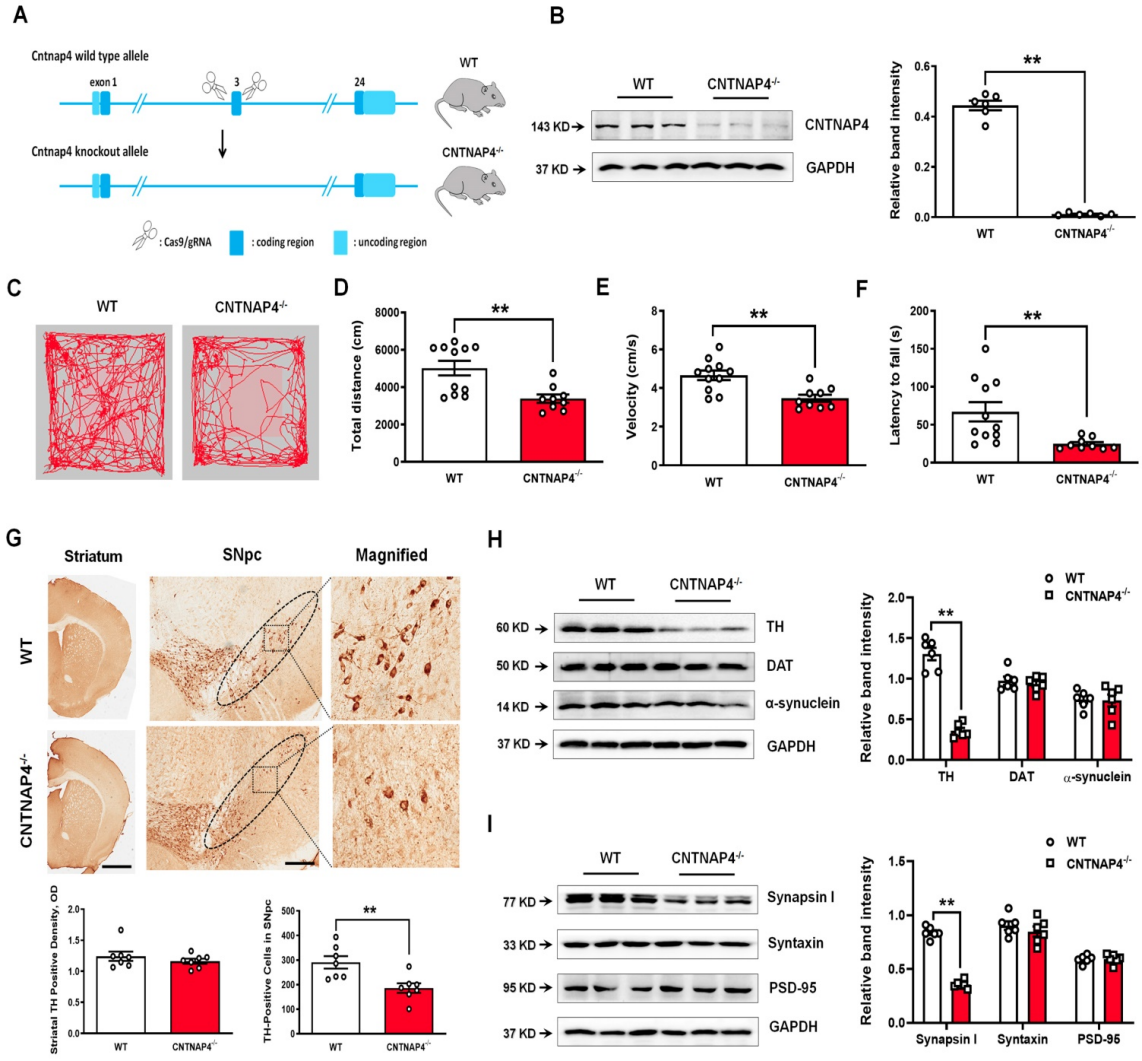
** CNTNAP4 knockout mice show movement deficits and nigral TH reduction**. (A) Strategy of establishing CNTNAP4 knockout mice. (B) Expression levels of CNTNAP4 in the midbrain of WT and CNTNAP4 knockout mice were determined by Western blotting. (C) Representative path tracings in the OFT. (D and E) Total distance travelled and movement speed in WT and CNTNAP4 knockout mice. (F) The rotarod test was used to examine the motor coordination of mice. (G) Immunohistochemical staining of TH-positive cells in the striatum and SNpc in WT and CNTNAP4 knockout mice (scale bars, 1 mm in striatum and 100 µm in SNpc). The ellipses in the middle column of panel G denote the boundaries of the SNpc, and the middle-column boxes denote the areas that are expanded in the right-hand columns in panel G. Quantification of striatal TH-positive density and TH-positive cells in the SNpc is shown below of panel G. (H and I) Expression levels of TH, DAT, α-synuclein, synapsin І, syntaxin, and PSD-95 in the midbrain of WT and CNTNAP4 knockout mice were determined by Western blotting. n = 11 for WT mice and n = 9 for CNTNAP4 knockout mice in Figure C-F. Western blotting results are from three of the six mice in each group. Results are expressed as the mean ± SEM. ^**^p < 0.01 vs. WT mice. Statistical significance was determined by Student's *t* tests.

**Figure 11:**
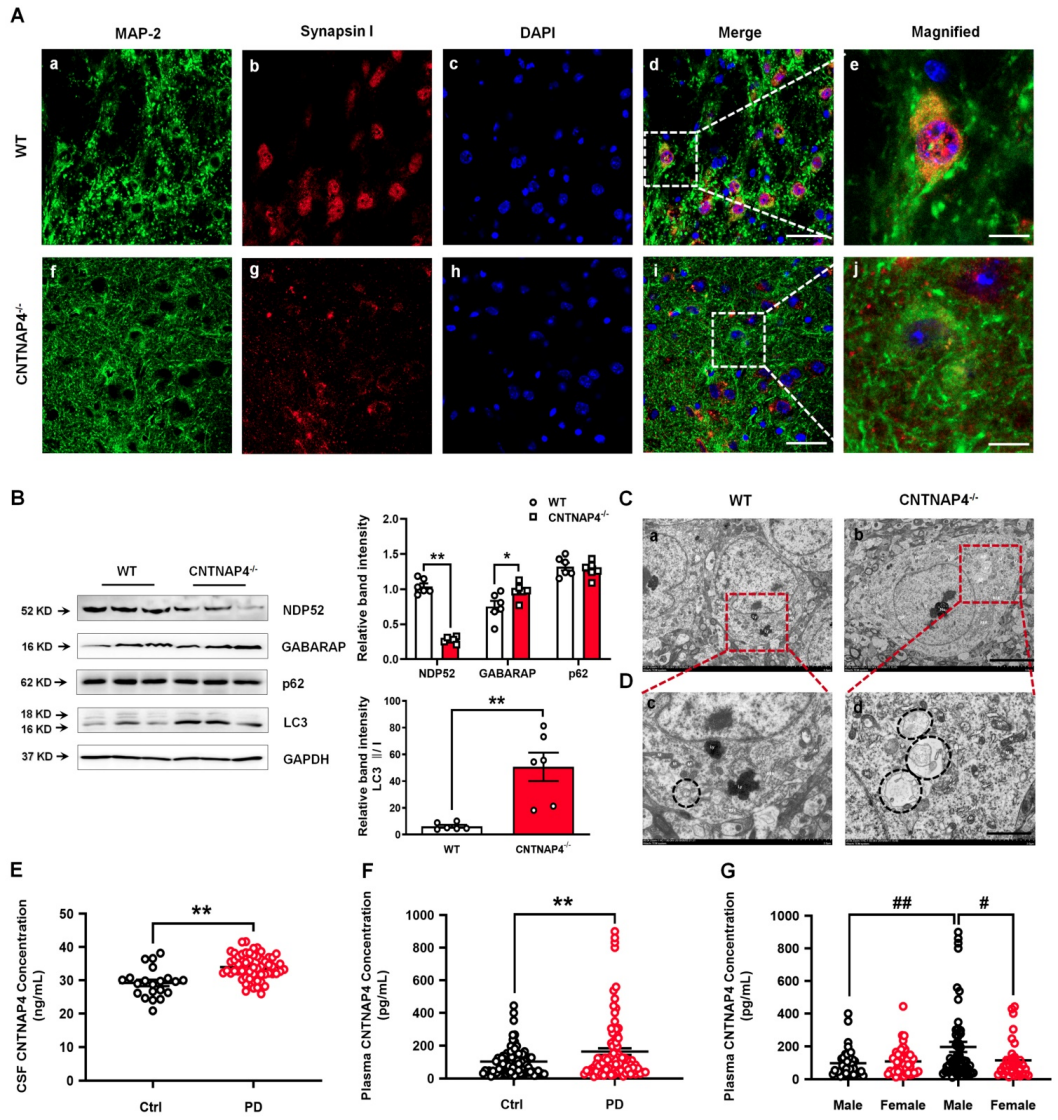
** Induced autophagy in CNTNAP4 knockout mice and increased CNTNAP4 concentrations in the CSF and plasma of PD patients.** (A) Immunofluorescence staining of MAP-2 and synapsin І in the SNpc of WT and CNTNAP4 knockout mice. Magnified images are expansions of boxed areas in corresponding panels in the left of each magnified image. (B) Expression levels of NDP52, GABARAP, p62, and LC3 in the midbrain of WT and CNTNAP4 knockout mice were determined by Western blotting. (C and D) Ultrastructural analysis of autophagosomes in the SNpc of WT and CNTNAP4 knockout mice. Magnified images in D are expansions of corresponding panels above in panel C. (E) CSF CNTNAP4 concentrations in controls and patients with PD were determined by ELISAs (n = 21 in control group and n = 58 in PD group). (F and G) Plasma CNTNAP4 concentrations in controls and patients with PD were determined by ELISAs (n = 90 in control group and n = 90 in PD group). Results are expressed as the mean ± SEM. Scale bar for A, 25 µm in the merged panel, 8 µm in the magnified panel; scale bar for C, 5 µm; scale bar for D, 2 µm. ^**^p < 0.01, ^*^p < 0.05 vs. WT mice or control group, and statistical significance was determined by a Student's *t*-test in B, E and F. ^##^*p* < 0.01, ^#^*p* < 0.05 vs. male PD group, and statistical significance was determined by a two-way ANOVA in G.

We performed genetic ablation of CNTNAP4 mice to explore its effect on motor function and DA neurons (strategy of establishing CNTNAP4 knockout mice is shown Figure 10A). We confirmed CNTNAP4 knockout by examining its protein level (Figure 10B). The results showed that the total distance traveled and movement speed in the OFT and latency to fall in the rotarod test were decreased in CNTNAP4 knockout mice compared to WT mice (Figure 10C-F). The behavioral results suggested that CNTNAP4 knockout mice showed movement disorder. We also found that TH expression was significantly decreased in the SNpc rather than the striatum in CNTNAP4 knockout mice compared with WT mice (Figure 10G). Consistently, the protein level of TH was decreased, while α-synuclein and DAT showed no obvious changes in CNTNAP4 knockout mice compared with WT mice (Figure 10H). We also found that synapsin І expression was significantly decreased in CNTNAP4 knockdown mice compared with WT mice (Figure 10I, 11A); this is consistent with our *in vitro* and *in vivo* models using CNTNAP4 AAV-shRNA virus. By contrast, there were no obvious changes in syntaxin and PSD-95 between WT and CNTNAP4 knockdown mice (Figure 10I). The protein levels of NDP52 were decreased, while the GABARAP and LC3-II/LC3-І ratio were increased (Figure 11B), in accordance with our *in vitro* CNTNAP4 knockdown results. TEM further confirmed that autophagy was induced in the SNpc in our CNTNAP4 knockout mice (Figure 11C-D).

### CSF and plasma CNTNAP4 concentrations are increased in patients with PD

Since we demonstrated the effects of CNTNAP4 deficiency in SNpc DA neurons on DA neuronal activity, α-synuclein expression, and dopaminergic synaptic transmission, we next examined whether CNTNAP4 concentrations are altered in human patients with PD. Here, for the first time, we detected CSF and plasma CNTNAP4 concentrations in human PD patients and found that CNTNAP4 expression in both was increased in PD patients (CSF, controls: 29.22 ± 4.39 ng/mL and PD patients: 33.98 ± 3.72 ng/mL; t = 4.779, df = 77, *P* < 0.0001; Figure 11E; plasma, controls: 103.20 ± 8.52 pg/mL and PD patients: 163.50 ± 20.30 pg/mL; t = 2.737, df = 178, *P* = 0.0068; Figure 11F). Furthermore, we also examined the differential expression of plasma CNTNAP4 in male and female individuals. Here we reported that the CNTNAP4 expression showed no obvious changes between male and female individuals in the control group (controls: male: 97.01 ± 13.17 pg/mL and female: 108.10 ± 11.23 pg/mL; *P* = 0.984; Figure 11G). However, CNTNAP4 concentration was increased in male PD patients as compared with male controls (male controls: 97.01 ± 13.17 pg/mL and male PD patients: 196.20 ± 30.70 pg/mL; *P* = 0.0072; Figure 11G), and female PD patients (PD patients: female: 114.30 ± 19.08 pg/mL and male: 196.20 ± 30.70 pg/mL; *P* = 0.0127; Figure 11G).

## Discussion

### CNTNAP4 deficiency induces DA neuronal degeneration

Mitochondria are key cellular organelles that provide ATP, and impaired mitochondrial function leads to a reduction in cellular energy levels and excessive reactive oxygen species (ROS) production, which are detrimental to DA neurons and are implicated in PD pathogenesis^34^, ^35^. In this study, according to our *in vitro* and *in vivo* results, we found that CNTNAP4 knockdown decreased the number of mitochondria (Figure 5E and 9B). We conclude that CNTNAP4 disrupted both mitochondrial structure and membrane potential, which may initiate mitochondrial stress, thus inducing mitophagy. Furthermore, we also indicated that CNTNAP4 knockdown decreased the expression of synapsin І, and we also found that CNTNAP4 deficiency reduced the number of presynaptic vesicles *in vivo*, which is consistent with the fact that CNTNAP4 is localized presynaptically and loss of CNTNAP4 results in synaptic deficits 18. These two facts may contribute to DA neuronal degeneration upon CNTNAP4 knockdown in SNpc. Importantly, we also confirmed nigral DA degeneration and decreased synapsin І expression in our CNTNAP4 knockout mice.

Although we found that CNTNAP4 knockdown in nigral DA neurons induced neuronal degeneration in mice, it did not affect TH or DAT expression in MN9D cells. We conclude that, as a member of the neurexin superfamily, CNTNAP4 has critical functions in neuronal communication 11. CNTNAP4 may affect TH expression via an indirect pathway, such as via neuron-neuron communication and synaptic transmission, which MN9D cells lack. Alternatively, although MN9D cells are considered as a suitable *in vitro* model to investigate DA neurons due to expressing TH, this model may not be able to recapitulate *in vivo* dopamine release and transport. This possible limitation may have influenced our CNTNAP4 results since CNTNAP4 has been reported to mainly affect midbrain dopaminergic release 18.

Our RNA-seq data showed that CNTNAP4 knockdown affected neuroactive ligand-receptor interactions via *Tacr2, Drd5, Nmur1, Calcrl,* and* Avpr2*, which is consistent with the role of CNTNAP4 in synaptic function. In addition, it is noteworthy that the DEGs included *Igf1, Nrg2* and* Ntf3*, which are also important in regulating DA synaptic transmission and neuron-neuron communication. Serum insulin-like growth factor 1 (IGF-1, encoded by *Igf1*) levels are correlated with frontal-lobe and striatal DA function and are implicated in dysfunctions in PD patients 36. *Neuregulin-2/Nrg2* ablation results in DA dysregulation and severe behavioral phenotypes related to psychiatric disorders 37. Further studies are needed to elucidate the potential correlation between CNTNAP4 and these genes. Intriguingly, our RNA-seq data also revealed that 11 DEGs were enriched in calcium-ion binding and 4 DEGs (*Tacr2, Drd5, Ryr1,* and* Cacna1c*) were enriched in calcium signaling pathways following CNTNAP4 knockdown. Since Ca^2+^ is required by mitochondria for the generation of ATP via the tricarboxylic acid cycle 38, we speculate that CNTNAP4 may affect mitochondrial function via regulating calcium signaling. Additionally, calcium signaling is also integral to neuronal activity and action potentials 39, 40, such as dopamine receptor D5 (encoded by* Drd5*) signaling from DA neuronal activity that modulates motor activity and striatal synaptic plasticity 41, 42. Moreover, ryanodine receptor 1 (encoded by* Ryr1*), poised for neuronal activity via Ca^2+^ entry, is critical for somatodendritic dopamine release 43, and genetic variations in *Cacna1c* (encoding Ca_v_1.2) are associated with PD risk 44. Thus, these DEGs being implicated in calcium signaling pathways following CNTNAP4 knockdown also provides further support for a role of CNTNAP4 in DA neuronal activity, in addition to mitochondrial function.

### CNTNAP4 knockdown in DA neurons increases α-synuclein expression

*Snca* (encoding α-synuclein) is a gene that increases the risk of developing PD, and α-synuclein aggregation is an important parkinsonian pathological phenotype and drug target 45. In this study, we found that CNTNAP4 knockdown increased α-synuclein expression both *in vitro* and *in vivo*. Although α-synuclein is a presynaptic protein distributed ubiquitously in the central nervous system, recent studies have suggested that it is mainly expressed in the substantia nigra GABAergic fibers 46, 47. In addition, regulation of GABA release through sulfonylurea receptor 1-regulated ATP-dependent potassium channels located on GABAergic neurons controls α-synuclein release via activation of the presynaptic GABA receptors 48. Since CNTNAP4 also regulates presynaptic GABA release, we conclude that CNTNAP4 affecting α-synuclein expression may be associated with its regulation of GABAergic transmission. Although these two proteins are located presynaptically, we did not find a direct interaction between CNTNAP4 and α-synuclein. As mentioned above, CNTNAP4 knockdown causes mitochondrial dysfunction and autophagy; thus, we concluded that the other mechanism by which CNTNAP4 knockdown increases α-synuclein expression may involve mitochondrial dysfunction and autophagy. Because α-synuclein is localized to mitochondria-associated ER membranes 49, mitochondrial dysfunction may induce α-synuclein aggregates, and, in turn, pathogenic α-synuclein aggregates preferentially bind to mitochondria and affect mitochondrial function 50, 51. Here, we found that CNTNAP4 knockdown reduced mitochondrial function, damaged mitochondria likely initiated mitophagy and promoted aberrant α-synuclein expression. Increased α-synuclein would consequently exacerbate mitochondrial damage and aggravated mitophagy further to clear α-synuclein and damage mitochondria. As such, increased α-synuclein expression may have been due to CNTNAP4 regulating GABAergic transmission, disrupting mitochondrial function and inducing mitophagy.

Our CNTNAP4 knockout mice showed movement deficits, nigral DA degeneration, and increased autophagy, which were consistent with our AAV-shRNA virus knockdown results. However, we did not find obvious reduced TH expression in the striatum in our CNTNAP4 knockout mice, and we conclude this may have resulted from adaptive mechanisms occurring in the striatum after nigral neuronal death. In addition, unlike the neurotoxins, MPTP and 6-OHDA, which specifically damage DA neurons, our CNTNAP4 knockout mice showed a delayed pattern of striatal DA neuron loss. In addition, we did not find increased α-synuclein expression in the SNpc in our CNTNAP4 knockout mice, suggesting that α-synuclein aggregation may exist in the older stage of these knockout mice. We may need to generate a dopaminergic CNTNAP4 ablation mice model in the future to clarify the specific effects of CNTNAP4 in DA neurons.

### CNTNAP4 deficiency may be linked with PD through regulating autophagy

Dysfunctional autophagy plays a vital role in the pathogenesis of PD 52, 53, and accumulation of autophagosomes or lysosomal depletion have been observed in the SNpc from postmortem brains of PD patients and in animal models 54, 55. However, there is still no report about whether CNTNAP4 could regulate autophagy.

In this study, according to our RNA sequencing data, we detected decreased expression of NDP52 upon CNTNAP4 knockdown. NDP52 is a key regulator of selective autophagy and NDP52 directs autophagic targets to autophagosomes by interacting with LC3 31, 32. Furthermore, LC3 and GABARAP amplify mitophagy via recruitment of NDP52 32. LC3 members (LC3A, LC3B and LC3C) and GABARAP subfamilies (GABARAP, GABARAPL1 and GABARAPL2) belong to the Atg8 family, and LC3/GABARAP proteins are known to regulate autophagosome biogenesis/maturation and cargo recognition. In addition, mislocalization of GABARAP could induce excessive p62 accumulation in autophagy-deficient neurons 56, suggesting its role in neuron autophagy. In this study, we found that CNTNAP4 knockdown increased while overexpression decreased the LC3-II/LC3-І ratio and GABARAP expression, suggesting that CNTNAP4 knockdown induced autophagy. Additionally, our CNTNAP4 knockout mice also had an increased autophagy level, as determined by examining the LC3-II/LC3-І ratio and nigral ultrastructural morphology. As stated above, CNTNAP4 knockdown caused mitochondrial dysfunction, and we presume that CNTNAP4 may induce mitophagy to promote the clearance of dysfunctional mitochondria. CNTNAP4 has been shown to interact with GABARAP to regulate GABAergic transmission in epilepsy 17, and GABARAP was originally identified as a GABA_A_ receptor-interacting protein and suggested to mediate the trafficking of GABA_A_ receptors 57. Since GABAergic signaling is linked to induction of autophagy via GABARAPL1 58, we conclude that CNTNAP4 deficiency induced autophagy may be also involved in regulating GABAergic signaling in DA neurons via GABARAP. However, further research is needed to address this hypothesis.

### CSF and plasma CNTNAP4 concentrations are increased in PD patients

In this study, for the first time, we report the CSF and plasma concentrations of CNTNAP4 in humans. Our clinical data indicated that CSF and plasma CNTNAP4 concentrations were increased in PD patients compared with control; particularly, plasma CNTNAP4 concentrations were increased in male but not female PD patients. In 2013, Iakoubov *et al*.^19^ reported that a common intronic CNV polymorphism in the *CNTNAP4* gene was associated with aging in females. In their study, the authors observed a positive association of the *CNTNAP4* R6782.1del/del variant with female but not male PD patients. Here, we further examined the function of CNTNAP4 in DA MN9D cells and found that CNTNAP4 knockdown in DA neurons may be relevant to PD pathogenesis. We reported increased CSF and plasma CNTNAP4 concentrations in PD patients and speculate that the increased plasma CNTNAP4 concentrations may have been due to the accelerated clearance of brain CNTNAP4 after DA neurons degenerate or from potential CNTNAP4 cleavage upon DA neuronal degeneration. Since CNTNAP4 possesses a large extracellular domain and functions as ErbB4 in interneurons 59, we hypothesize that it may share a similar cleavage process or signaling pathway with ErbB4 59, 60, which produces an extracellular fragment and a CNTNAP4-intracellular domain. In PD, some stimuli may accelerate the cleavage process, damage normal CNTNAP4 function, and release extracellular fragments into the cerebrospinal fluid or plasma. Thus, circulating CNTNAP4 may also represent a biomarker for diagnosing PD patients. Regarding the unchanged plasma CNTNAP4 concentration in female PD patients as compared with their controls, we hypothesize that estrogen may play a certain role in regulating CNTNAP4 in the pathogenesis of PD.

## Conclusion

In this study, for the first time, we elucidated a novel role of CNTNAP4 in DA neurons *in vitro* and *in vivo*, and that CNTNAP4 deficiency may initiate pathological phenotypes relevant to PD. We found that CNTNAP4 deficiency in DA neurons induced DA neuronal degeneration, increased α-synuclein expression, increased mitophagy, and disrupting expression of synaptic proteins. Furthermore, we reported that CSF and plasma CNTNAP4 expression was increased in PD patients. Our findings support that CNTNAP4 may be a novel biomarker and drug target for PD.

## Supplementary Material

Supplementary figures.

## Abbreviations

AAV: adeno-associated virus
Atg8: autophagy-related 8
Calcoco2: calcium binding and coiled-coil domain 2
CNTNAP4: Contactin-associated protein-like 4
CSF: cerebrospinal fluid
DA: dopaminergic
DAT: dopamine transporter
GABA_A_Rs: GABA type-A receptors
GABARAP: GABA_A_R-associated protein
LC3: light chain 3
MMP: mitochondrial membrane potential
MPP^+^: 1-methyl-4-phenylpyridinium
MPTP: 1-methyl-4-phenyl-1,2,3,6-tetrahydropyridine
shRNA: short hairpin RNA
siRNAs: small interfering RNAs
SN: substantia nigra
SNpc: substantia nigra pars compacta
SQSTM1: sequestosome 1
TH: tyrosine hydroxylase
