# Supplementary material for: CNTNAP4 deficiency in dopaminergic neurons initiates parkinsonian phenotypes
Source: Theranostics. 2020 Feb 10;10(7):3000–21. doi: 10.7150/thno.40798 (PMC7053186; doi:10.7150/thno.40798)
Supplement: Supplementary file 1 — Supplementary figures. [file thnov10p3000s1.pdf]

Supplementary Materials

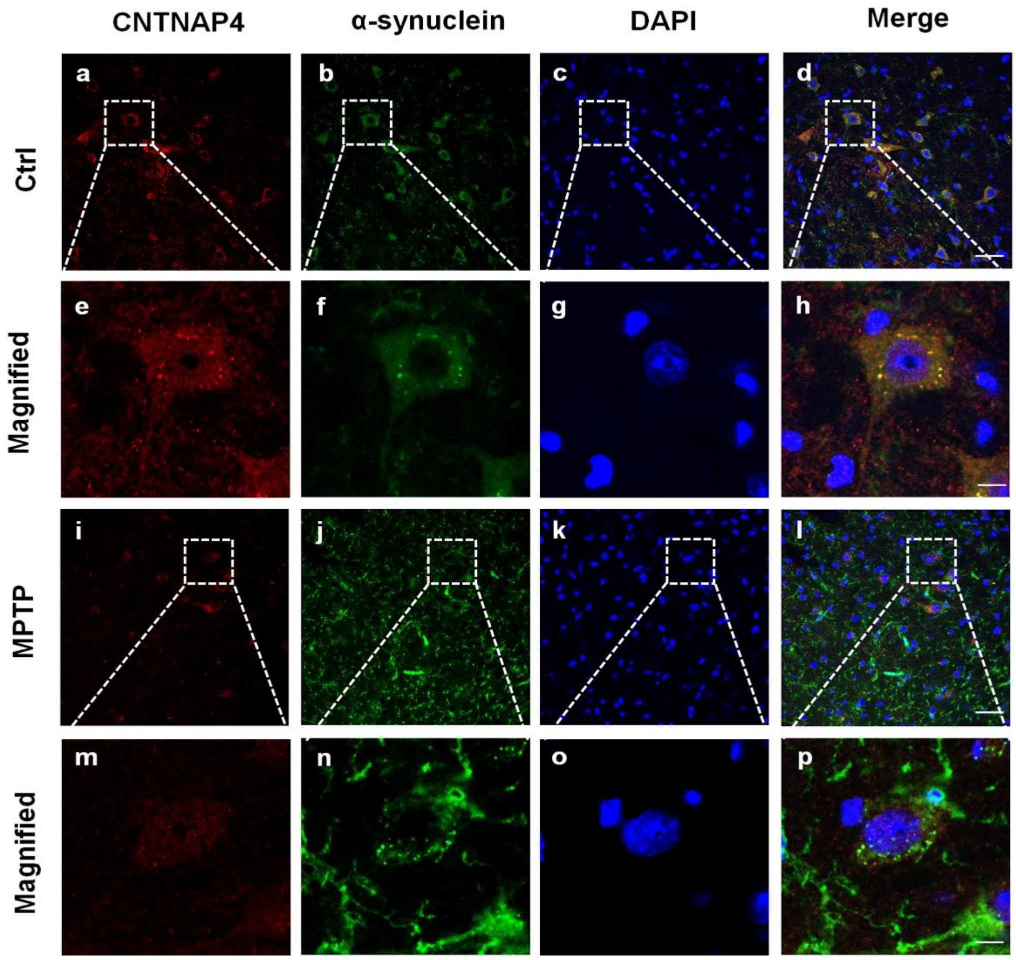

Figure S1. CNTNAP4 expression is decreased in the chronic MPTP mouse model. Immunofluorescent staining of CNTNAP4 and α-synuclein in the SNpc of control and chronic MPTP mice. Boxes denote areas that are magnified in the columns labeled as "Magnified" (scale bars, 25 µm in A–D and I–L; 5 µm in E–H and M–P).

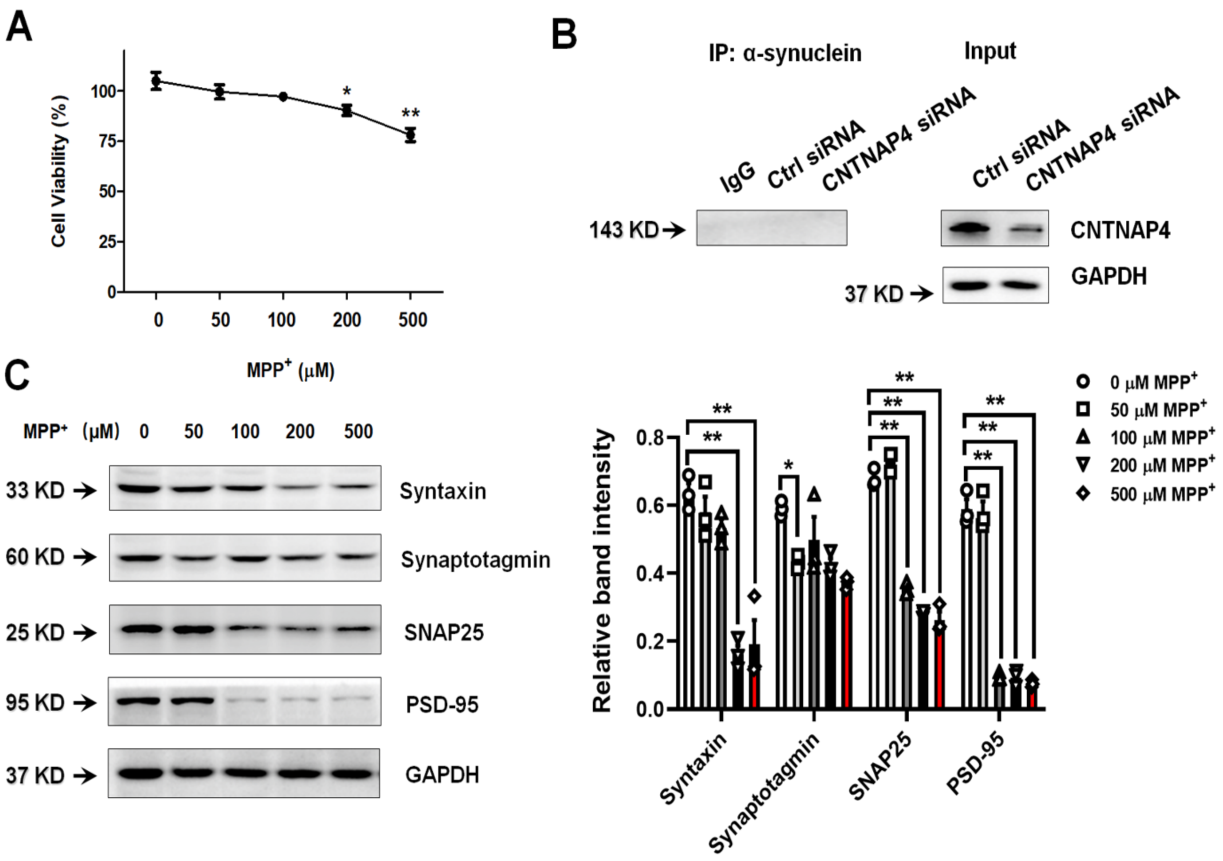

Figure S2. Effect of MPP$^+$ on synaptic proteins in MN9D cells. (A) Effects of different concentrations of MPP$^+$ on the viability of MN9D cells. (B) Immunoprecipitation did not detect an interaction between CNTNAP4 and α-synuclein in MN9D cells. (C) Effects of different concentrations of MPP$^+$ on expression levels of syntaxin, synaptotagmin, SNAP25, and PSD-95 were determined by Western blotting (n = 3 per group). $^{**}p < 0.01$ vs. control group. Statistical significance was determined by one-way ANOVAs and Tukey tests for *post-hoc* comparisons.

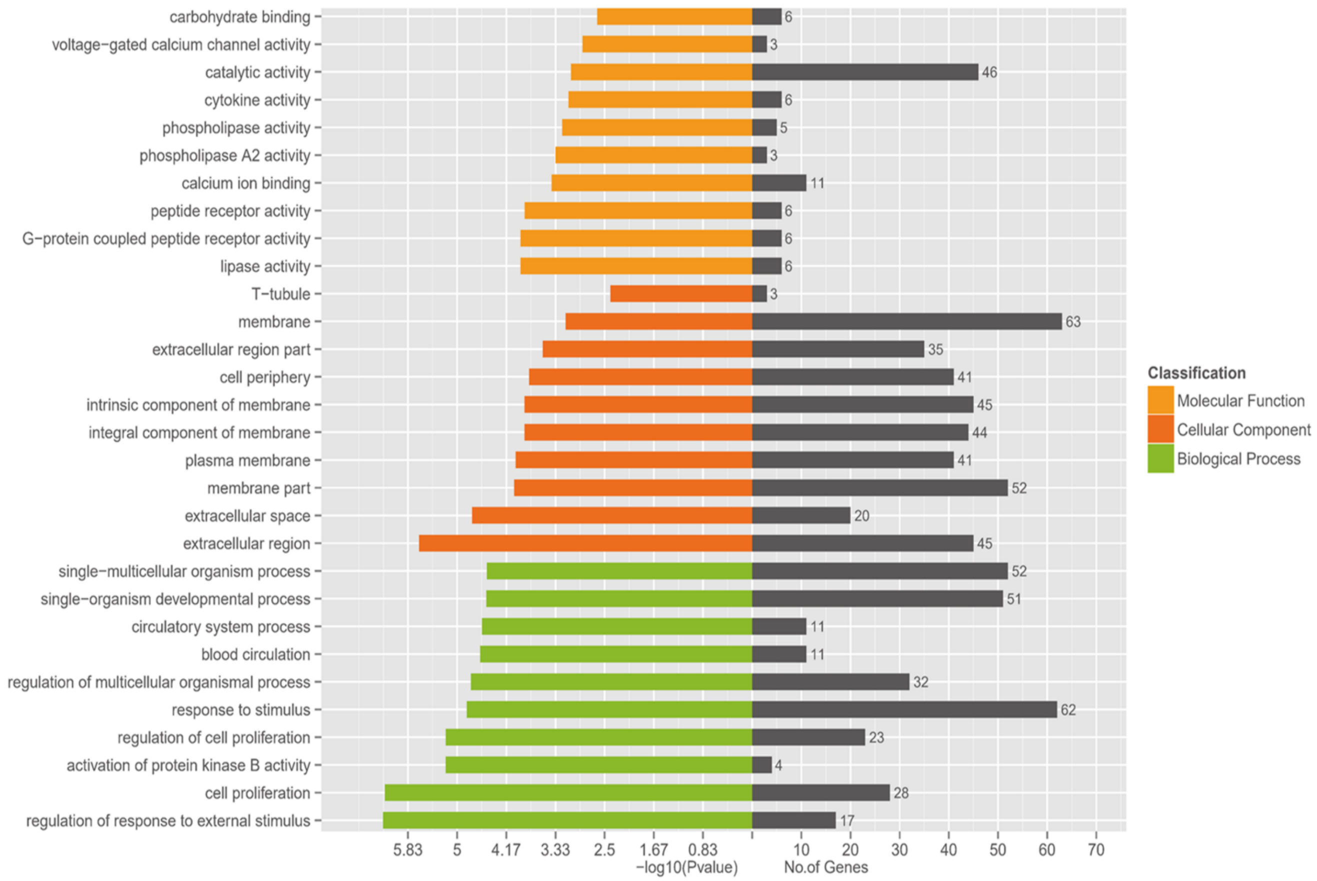

Figure S3. GO analysis of DEGs following CNTNAP4 knockdown in MN9D cells. After transfection with CNTNAP4 siRNA for 72 h, transcriptomic analysis was performed by RNA-seq. GO analysis was used to determine the functions of DEGs following CNTNAP4 knockdown in MN9D cells.

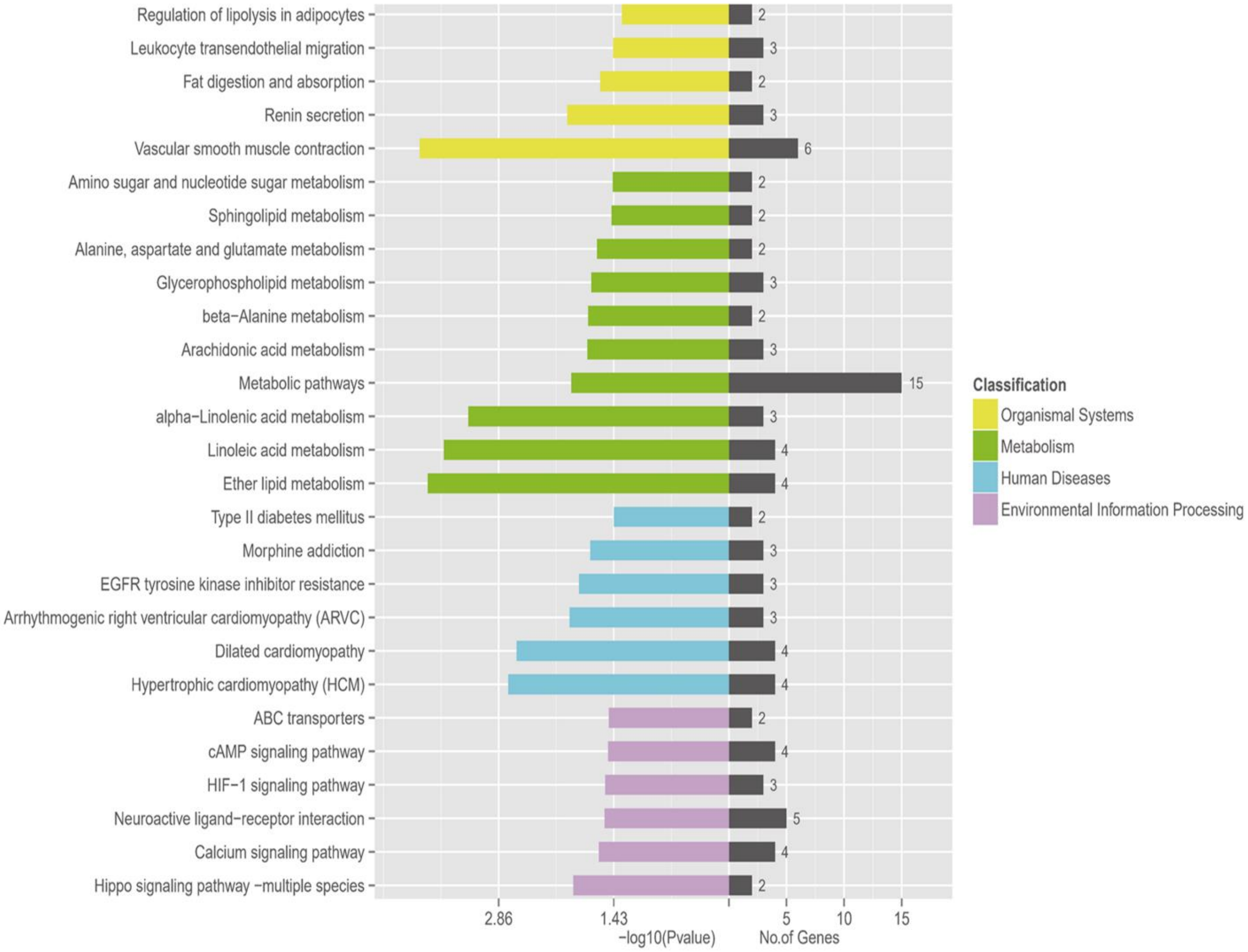

Figure S4. KEGG analysis of DEGs following CNTNAP4 knockdown in MN9D cells. After transfection with CNTNAP4 siRNA for 72 h, transcriptomic analysis was performed by RNA-seq. KEGG analysis was used to determine the functions of DEGs following CNTNAP4 knockdown in MN9D cells.

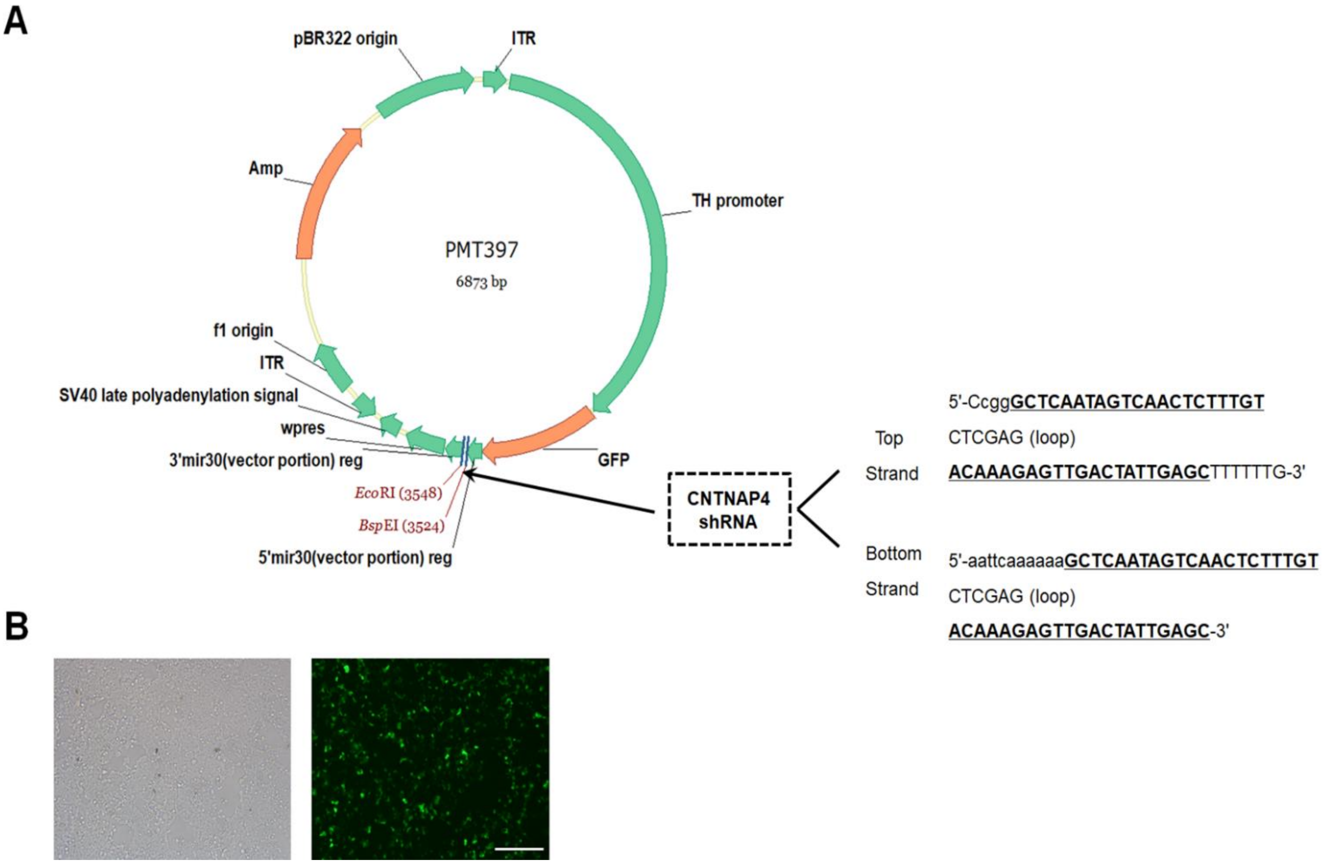

Figure S5. Design of CNTNAP4 AAV-shRNA. (A) AAV2/9-TH-3Flag-miR30-GFP-polyA (pMT397) shRNA was the interfering vector used in this study. The CNTNAP4 AAV-shRNA and Ctrl AAV-shRNA were generated by ligating annealed oligonucleotides encoding sh CNTNAP4 or a scramble sequence into the *Bsp* EI/*Eco* RI site of the AAV2/9-TH-3Flag-miR30-GFP-polyA (pMT397) shRNA vector. CNTNAP4 AAV-shRNA was constructed to express shRNA targeting CNTNAP4 (GCTCAATAGTCAACTCTTTGT) with the TH promoter. (B) The infection efficiencies of different titers of CNTNAP4 AAV-shRNA were examined in HEK-293T cells.

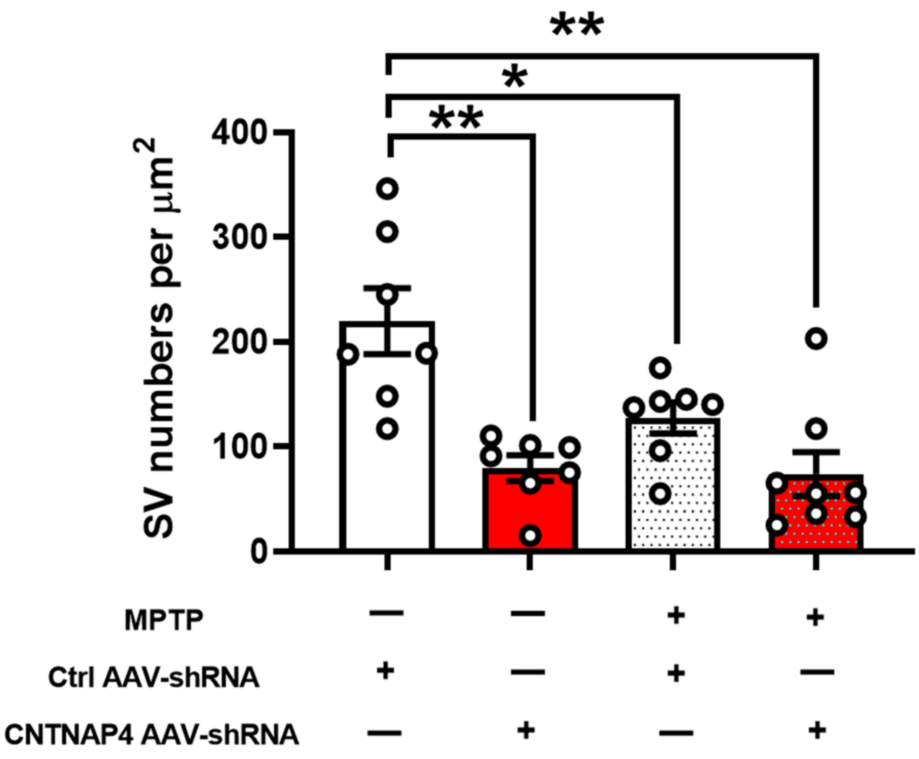

Figure S6. CNTNAP4 knockdown in SNpc DA neurons reduces synaptic vesicles in the SNpc. Mice received Ctrl AAV-shRNA or CNTNAP4 AAV-shRNA in the SNpc, and then they were administrated saline or MPTP. The ultrastructure in the SNpc was examined by TEM, and quantification of synaptic vesicles is shown (n = 7 in each of the control, CNTNAP4 AAV-shRNA, and MPTP group; n = 8 in the MPTP+CNTNAP4 siRNA group). Results are expressed as the mean ± SEM. $^{**}p < 0.01$, $^{*}p < 0.05$ vs. Control group. In the figure, SV denotes synaptic vesicles. Statistical significance was determined by one-way ANOVAs and Tukey tests for *post-hoc* comparisons.
